# Folate-receptor-targeted co-self-assembly carrier-free gemcitabine nanoparticles loading indocyanine green for chemo-photothermal therapy

**DOI:** 10.3389/fbioe.2023.1266652

**Published:** 2023-09-21

**Authors:** Shuang Kuang, Shuhan Liu, Shiyu Wang, Liang Yang, Yingchun Zeng, Xin Ming

**Affiliations:** Study on the Structure-Specific Small Molecular Drug in Sichuan Province College Key Laboratory, School of Pharmacy, Chengdu Medical College, Chengdu, Sichuan, China

**Keywords:** co-self-assembly, carrier-free, gemcitabine, indocyanine green, chemophotothermal therapy

## Abstract

The carrier-free chemo-photothermal therapy has become a promising strategy to improve anti-cancer therapeutic efficacy owing to the combination of chemotherapy and photothermal therapy, with improved chemotherapy drug pharmacodynamics and pharmacokinetics, high drug loading, and reduced toxicity. We designed a novel carrier-free targeting nanoparticles, co-self-assembled amphiphilic prodrugs 3′,5′-dioleoyl gemcitabine (DOG), and tumor-targeted γ-octadecyl folate (MOFA), with encapsulated US Food and Drug Administration (FDA)-approved photosensitizer indocyanine green (ICG) for synergistic chemo-photothermal therapy. The DOG linking oleic acid to the sugar moiety of gemcitabine (GEM) showed better self-assembly ability among GEM amphiphilic prodrugs linking different fatty acids. The readily available and highly reproducible 3′,5′-dioleoyl gemcitabine/γ-octadecyl folate/indocyanine green (DOG/MOFA/ICG) nanoparticles were prepared by reprecipitation and showed nano-scale structure with mono-dispersity, great encapsulation efficiency of ICG (approximately 74%), acid- and laser irradiation-triggered GEM release *in vitro* and sustained GEM release *in vivo* after intravenous administration as well as excellent temperature conversion (57.0°C) with near-infrared laser irradiation. The combinational DOG/MOFA/ICG nanoparticles with near-infrared laser irradiation showed better anti-tumor efficacy than individual chemotherapy or photothermal therapy, with very low hemolysis and inappreciable toxicity for L929 cells. This co-self-assembly of the ICG and the chemotherapy drug (GEM) provides a novel tactic for the rational design of multifunctional nanosystems for targeting drug delivery and theranostics.

## 1 Introduction

Cancer-related deaths are becoming a major global health problem (Siegel et al., 2021). Lung cancer is the second most frequently diagnosed malignant tumor, and a majority of primary lung cancers arise from non-small cell lung cancer (NSCLC) (Duma et al., 2019). Nucleoside analog GEM exerting anticancer ability by interfering with DNA synthesis and inhibiting nucleotide reductase activity has been approved by the FDA for treating NSCLC (Abdayem et al., 2014; Shelton et al., 2016; Li et al., 2020c). However, GEM’s clinical applicability is hampered by its instability and short plasma half-life (Maiti et al., 2013; Zhang et al., 2017; Abdel-Rahman et al., 2018). Additionally, the rapid development of drug resistance makes a single agent unable to achieve satisfactory treatment in cancer cells (Xiao et al., 2012). As an alternative approach, the co-delivery of multifunctional nanoagents is promising for overcoming these problems and enhancing anticancer drug efficacy (Al-Lazikani et al., 2012; Meng et al., 2015; Li et al., 2022). It has been demonstrated that the co-delivery of photothermal agents and chemotherapeutic drugs can show more sound therapeutic effects than the delivery of a single agent (Li et al., 2020b; Liang et al., 2020; Huang et al., 2021). Tricarbocyanine dye ICG approved for clinical use by the FDA as a photothermal therapy dye can cause thermal ablation in tumor tissues by generating heat within near-infrared (NIR) laser irradiation (Wang et al., 2017b; Ji et al., 2022). In addition, it may alter the permeability of cell membranes to accelerate drug release and synergistically enhance the drug cytotoxicity to cancer cells. For example, a multicomponent lipid nanoparticle which was made up of doxorubicin (DOX) attached ICG-loaded liposomes and carbon dots co-delivered chemotherapeutic drug DOX and ICG via carbon dots carrier. It significantly increased tumor cell apoptosis and death by enhancing tumor cell uptake of nanoparticles, elevating tumor site temperature and rapidly releasing DOX (Xue et al., 2018).

Until recently, co-delivery of multifunctional drugs has been made in various nano-sized carriers for encapsulating multiple agents and delivering them to cancer cells (Liang et al., 2017; Cheng et al., 2018; Ghitman et al., 2020). These nano-sized carriers are favorable to optimizing drug pharmacokinetics, increasing accumulation of drugs at tumor sites by the permeability and retention (EPR) effect or active target, thereby boosting the efficiency of treatment (Li et al., 2020a; Sandhiutami et al., 2020; Shakeran et al., 2021). However, it is difficult for chemotherapy drugs to attain antitumor effects only via passive targeting of the EPR effect of nanosystems. Thus, the active targeting ligands (such as cell-penetrating peptides, small molecules, or membranes from host cells) are used to improve tumor targeting (Cao et al., 2016; Izci et al., 2021). The folate receptor is commonly overexpressed on various tumor types (including NSCLC) and frequently used for active targeted nanosystems delivery (Santra et al., 2011). Besides, the main moiety in most drug delivery systems is carriers (the weight ratio is typically more than 90%), which makes the capacity for loading drugs generally limited (Ekladious et al., 2019; Li et al., 2021). In addition, the presence of inert carriers would increase the systemic toxicity of drug formulation and generate additional burdens to patients by their subsequent degradation (Milosevic et al., 2020; Kihara et al., 2021; Stater et al., 2021). As an alternative, carrier-free nanomedicines entirely made up of one or more drugs have more advantages in tumor theranostic due to high drug loading, low toxicity, and improved pharmacodynamics and pharmacokinetics (Liu and Zhang, 2020; Huang et al., 2021; Karaosmanoglu et al., 2021). Furthermore, in response to cues in the tumor microenvironment, certain carrier-free nanoparticles can undergo morphological alteration, changing their sizes and increasing drug retention and penetration in the tissue (Yang et al., 2019; Zhang et al., 2020).

**FIGURE 1:**
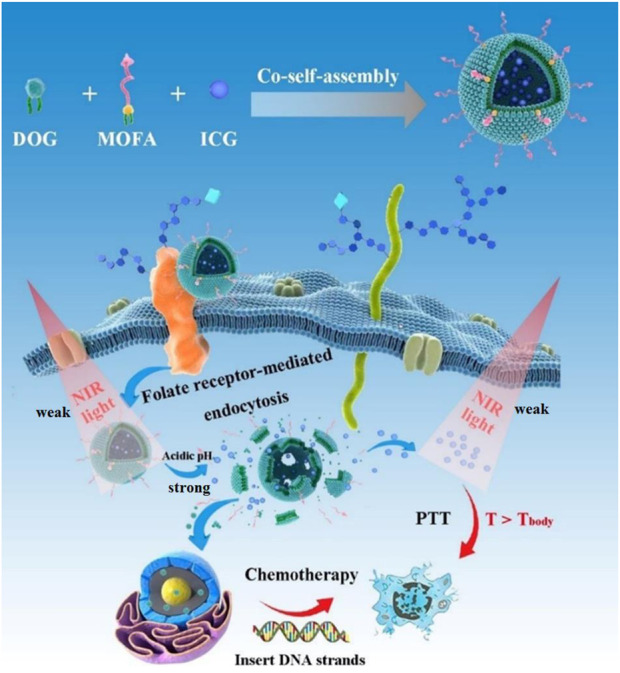
Schematic diagram of the preparation of DOG/MOFA/ICG nanoparticles and their action.

Here, we developed carrier-free functionalized nanoparticles (DOG/MOFA/ICG nanoparticles) for chemo-photothermal therapy to improve the pharmacokinetics of GEM and enhance cell lethality. As shown in Figure 1, the carrier-free functionalized DOG/MOFA/ICG nanoparticles were prepared by co-self-assembly of amphiphilic anticancer drug agent (DOG), active targeting agent (MOFA), and encapsulated photothermal therapy agent (ICG). After efficient internalization, the DOG prodrug would respond to the acidic tumor microenvironment and release GEM and ICG due to the cleavage of ester bonds. Then, GEM could enter the nucleus to exert an anti-tumor effect, and ICG could convert 808 nm laser irradiation energy into heat for photothermal therapy. These results highlight the potential of functionalized nanoparticles for highly efficient synergistic chemo-photothermal therapy for cancer inhibition, as well as providing a novel method for the theranostics nanosystems.

## 2 Materials and methods

### 2.1 Materials

All materials, solvents, and intermediates were purchased from commercial suppliers and were used without further purification. GEM, ICG, folate (or folic acid, FA), oleic acid, linoleic acid, myristic acid, decanoic acid, heptanoic acid, 1-ethyl-3-(3-dimethylaminopropyl) carbodiimide hydrochloride (EDCI), 4-dimethylaminopyridine (DMAP), 1-octadecanol and pyrene were purchased from Leyan (Shanghai, China). Cell Counting Kit-8 (CCK-8), Annexin V-FITC Apoptosis Detection Kit, 4′,6-diamino-2-phenylindole (DAPI) were purchased from Beyotime Bio-Technology Co., Ltd. (Shanghai, China). Fetal bovine serum (FBS) was purchased from Gibco (Thermo Fisher Scientific, United States). Dulbecco’s Modified Eagle Medium (DMEM), trypsin, penicillin/streptomycin solution were obtained from Hyclone (Logan, United States). A549 cancer cells (a human non-small cell lung carcinoma cell line) and L929 normal cells (a mouse fibroblasts cell line) were purchased from the American Type Culture Collection (ATCC, Rockville, MD). ^1^H NMR spectra were recorded on a 400 MHz instrument (Bruker, Germany) with TMS as an internal standard. MS experiments were performed on a micro TOF-Q II (Bruker, Germany), employing electrospray ionization in positive mode.

### 2.2 Animals

Kunming mice (4–6 weeks old, 18–22 g weight) were purchased from Chengdu Dashuo Biological Institute (Chengdu, China). All animal experiments complied with the Animal Management Rules of the Ministry of Health of the People’s Republic of China. *In vivo* studies were approved by the Institutional Animal Care and Use Committee (no. 2023-011) at Chengdu Medical College.

### 2.3 Synthesis of amphiphilic prodrugs

**FIGURE 2:**
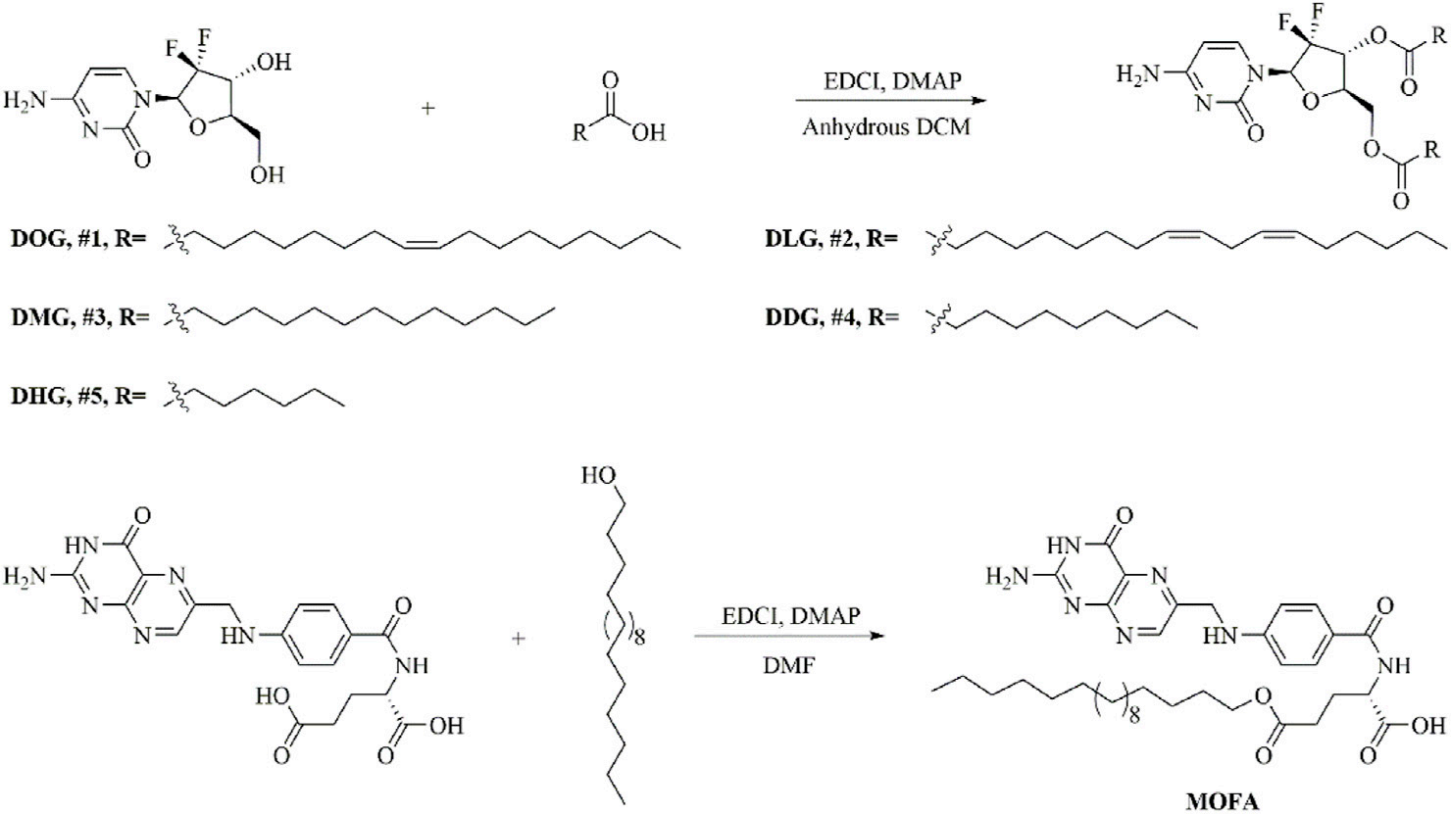
The synthesis route of amphiphilic prodrugs and γ-octadecyl folate (MOFA).

General procedure for the synthesis of GEM amphiphilic prodrugs: The synthesis routes of amphiphilic prodrugs and MOFA are shown in Figure 2. Briefly, GEM (1.00 g, 3.80 mmol) was suspended in dichloromethane (25.0 mL). EDCI (1.75 g, 9.12 mmol), DMAP (0.56 g, 4.56 mmol), and fatty acid (9.12 mmol, 2.4 eq) were added. The reaction mixture was stirred at room temperature for 24 h. The reaction was monitored by thin-layer chromatography (TLC). After completion, the reaction mixture was washed twice with water, and dried over Na_2_SO_4_. The organic layer was evaporated to get the residue which was purified by column chromatography using petroleum ether: ethyl acetate (5:1) to get pure products.

3′,5′-Dioleoyl gemcitabine (DOG, **#1**): GEM was reacted with oleic acid.
3′,5′-Dilinoleoyl gemcitabine (DLG, **#2**): GEM was reacted with linoleic acid.
3′,5′-Dimyristoyl gemcitabine (DMG, **#3**): GEM was reacted with myristic acid.
3′,5′-Didecanoyl gemcitabine (DDG, **#4**): GEM was reacted with decanoic acid.
3′,5′-Diheptanoyl gemcitabine (DHG, **#5**): GEM was reacted with heptanoic acid.

Synthesis of γ-octadecyl folate (MOFA): FA (300.0 mg, 0.680 mmol) was dissolved in 15 mL of N, N-dimethylformamide (DMF), 1-octadecanol (138.9 mg, 0.680 mmol), EDCI (260.6 mg, 1.360 mmol) and DMAP (33.2 mg, 0.272 mmol) were added. After stirring at room temperature for 48 h under argon, the reaction mixture was poured into 60 mL of acetone and stirred for 30 min to afford precipitate, which was filtrated, washed with acetone, and dried under vacuum.

### 2.4 Screening for self-assembly ability of amphiphilic GEM prodrugs

The different self-assembled nanoparticles were prepared by reprecipitation-dialysis method (Wang et al., 2017). Briefly, 24.0 mg amphiphilic GEM prodrugs were respectively dissolved in 4 mL of DMF, dimethylsulfoxide (DMSO), methanol (MeOH), and ethanol (EtOH), and kept stirring for 4 h. Subsequently, the above solution was very slowly dropwise added to 20 mL of ultra-pure water and vigorously stirred for another 2 h. Next, the obtained solution was transferred into a dialysis bag (molecular weight cut-off = 3.5 kDa) and dialyzed in ultra-pure water for 24 h, and the water was replaced regularly (4 h). DOG, DLG, DHG, DDG, and DMG nanoparticles were prepared according to the above method.

Next, a series of characterizations were implemented on the obtained DOG, DLG, DHG, DDG, and DMG nanoparticles. The zeta potential, size distribution, and polydispersity index (PDI) of obtained different nanoparticles were measured by laser particle size analyzer (Malvern Nano ZS90, Malvern, United Kingdom).

### 2.5 Preparation and characterization of DOG, DOG/MOFA, DOG/ICG, and DOG/MOFA/ICG nanoparticles

DOG, DOG/MOFA, DOG/ICG, and DOG/MOFA/ICG nanoparticles were prepared according to the above screening results. Typically, DOG (24.0 mg); DOG (24.0 mg), and MOFA (2.4 mg); DOG (24.0 mg) and ICG (2.4 mg); DOG (24.0 mg), MOFA (2.4 mg) and ICG (2.4 mg) were respectively dissolved in 4 mL of DMSO in a round-bottomed flask and kept stirring for 4 h. The above solutions were slowly added into 20 mL of ultra-pure water and vigorously stirred for another 2 h. The obtained solutions were transferred into a 3.5 kDa molecular weight cut-off dialysis bag and dialyzed in ultra-pure water for 24 h, and the water was replaced every 4 h.

Next, a series of characterizations on the obtained DOG, DOG/MOFA, DOG/ICG, and DOG/MOFA/ICG nanoparticles were performed. The zeta potential, size distribution, and PDI of obtained nanoparticles were analyzed by laser particle size analyzer. Transmission electron microscopy (TEM) was conducted on an HT7700 transmission electron microscope (Hitachi, Japan). The UV data of different nanoparticles was measured by a Specord^®^ 50 Plus UV-vis spectrophotometer (Analytik Jena, Germany). The fluorescence spectrum was implemented on the RF-6000 Spectro fluorometer (Shimadzu, Japan).

### 2.6 Assembly mechanisms

The assembly mechanisms of DOG/MOFA/ICG nanoparticles were investigated using computer simulations. Molecular dynamics (MD) calculations were carried out using the Discovery Studio software based on the CharMM (Chemistry at Harvard Macromolecular Mechanics). In the simulation phase, the dielectric constant was 80 (simulating an aqueous environment), using a leapfrog Verlet dynamics integrator. In the first step, the energy minimization was performed using the steepest descent method (maximum step: 100, RMS gradient: 1.0). In the heating phase we simulated 50 ps (time step: 2 ps) at a target temperature of 300 K. The equilibrium phase was run for 100 ps to keep the temperature at 300 K. In the production step, 1,000,000 ps were run and the results were saved in 1,000 ps intervals (snapshots). The Fourier Transform infrared (FT-IR) spectrum was performed on a Thermo Fisher Nicolet Is5 infrared spectrometer (Thermo Fisher Scientific, Waltham, MA, United States).

### 2.7 Loading and drug release

The quantitative analysis of GEM, DOG, and ICG was obtained by establishing a standard curve through Specord^®^ 50 Plus UV-vis spectrophotometer. Methanol was added into 1 mL of self-assembled nanoparticles for ultrasonic emulsification. The absorbance was determined at 784 nm (ICG), with blank correction. A summary method for calculating drug loading (DL) and entrapment efficiency (EE) was shown as follows.

$$EE\;\left(\%\right)=\frac{amount\;of\;DOG\;or\;ICG\;in\;self-assembled\;nanoparticles}{amount\;of\;DOG\;or\;ICG\;added}\times 100$$ (1)

$$DL\;\left(\%\right)=\frac{amount\;of\;DOG\;or\;ICG\;in\;self-assembled\;nanoparticles}{amount\;of\;co-self-assembled\;nanoparticles}\times 100$$ (2)

The cumulative release of GEM from different nanoparticles was studied by the dialysis method to investigate the release behavior of different nanoparticles. At first, a total of 0.8 mL of different nanoparticle solutions were transferred to a dialysis bag (molecular weight cut-off = 3.5 kDa) and immersed in 30 mL PBS at pH 7.4, 6.5, and 5.5. The controlled release of the nanoparticles in the absence/presence of esterase enzyme was examined. The mixture solutions were gently shaken in a constant-temperature incubator (37°C, 100 rpm). The dialysate (0.5 mL) in dialysis tubes was withdrawn at each predetermined time and replaced with fresh PBS with the same pH. Secondly, the release content of GEM and ICG was measured with the UV-vis spectrometer (λ_GEM_ = 320 nm, λ_ICG_ = 784 nm). In addition, to investigate the influence of the laser irradiation on the release behavior of DOG/ICG and DOG/MOFA/ICG nanoparticles, *in vitro* drug release experiments were also implemented in the same way (laser irradiated with 808 nm, 1 W/cm^2^).

### 2.8 *In vitro* physical stability

The stability of different nanoparticles in different media was examined, 1 mL of different nanoparticles were respectively mixed with 1 mL DMEM, PBS (pH 7.4). For the stability of different nanoparticles in FBS, 900 μL different nanoparticles and 100 μL FBS were respectively mixed. Then, the PDI and size of nanoparticles in various mediums were examined for 7 days, respectively. Moreover, the stability of different nanoparticles under laser irradiation was studied, 1 mL of different nanoparticles were respectively transferred to EP tubes and irradiated by the 808 nm laser (VCL-808 nm M 0–2 W, Beijing Honglan Electronic Technology Co., Ltd., Beijing, China) with a power density of 1 W/cm^2^ for 5 min. Subsequently, the nanoparticle solution was cooled to room temperature and the size distribution was measured, and this process was cycled three times.

### 2.9 Determination of critical micelle concentration (CMC)

The CMC of different nanoparticles was measured using a pyrene probe as a fluorescence probe (Cabral et al., 2018). Briefly, the different nanoparticles were respectively diluted by ultra-pure water to different concentrations (0.0003125–50 μM) and mixed with 1 mM pyrene in acetone. Then equilibrating at room temperature for 4 h, the fluorescence intensity of the above mixture solutions was determined (λ_ex_ = 339 nm, λ_em_ = 350–500 nm) using an RF-6000 Spectro fluorometer (Shimadzu, Japan). The intensity ratios of the first (371 nm) to the third (381 nm) peaks (I1/I3) were plotted with the copolymer concentration’s logarithm. The intersection of the two lines was the CMC value.

### 2.10 *In vitro* photothermal effect

The 808 nm laser was exploited in the photothermal effect experiments. Ultra-pure water, free ICG (10 μg/mL), and different nanoparticles (calculated as 10 μg/mL by ICG) were respectively transferred to EP tubes and irradiated by the 808 nm laser with 1 W/cm^2^ for 5 min. The temperature change was monitored by a Fluke TiS20 thermal infrared imaging camera (Washington, United States).

### 2.11 Hemolysis assay

A fresh rabbit blood sample (2.0 mL) was stabilized by heparin sodium and then mixed with 4.0 mL of PBS (4°C). After centrifugation at 3,000 rpm for 5 min, the red blood cells (RBCs) were washed 3 times with cold PBS (4°C) by centrifugation-redispersion until the supernatant was clarified. Subsequently, the different nanoparticles were incubated with 2% (w/v) of RBCs dispersions at 37°C for 6 h with various concentrations. Further, PBS was used as a negative control (NC), and ultra-pure water was used as a positive control (PC). Finally, the samples were centrifugated at 4,000 rpm for 10 min, the supernatants were collected and the released hemoglobin was determined with a UV-vis spectrometer at 540 nm.

$$Hemolysis\;\left(\%\right)=\frac{A\left(\mathrm{sample}\right)-A\left(\mathrm{negative}\right)}{A\left(\mathrm{positive}\right)-A\left(\mathrm{negative}\right)}\times 100$$ (3)

Where A_(sample)_, A_(negative)_, and A_(positive)_ are the absorbance values of the sample group, the negative control group, and the positive control group, respectively.

### 2.12 Cell culture

A549 cancer cells (a human NSCLS cell line) and L929 normal cells (a mouse fibroblasts cell line) were all cultured in DMEM supplemented with 10% FBS and 1% penicillin/streptomycin at 37°C in a humidified atmosphere containing 5% CO_2_.

### 2.13 Cellular uptake of DOG/ICG and DOG/MOFA/ICG nanoparticles

The experiments on cellular uptake were performed on fluorescence microscopy and flow cytometry. A549 cells were seeded in 24-well plates at 1 × 10^5^ cells per well in 0.5 mL DMEM and cultured for 24 h. Subsequently, 0.5 mL DMEM containing ICG, DOG/ICG, and DOG/MOFA/ICG nanoparticles with the same ICG concentration (10 μg/mL) was added to every well. The cells were further incubated at 37°C for 1 and 4 h, respectively. Thereafter, the cells were washed with cold PBS three times and fixed with 4% paraformaldehyde for 30 min at room temperature. After that, the cells were washed with cold PBS three times and stained with DAPI for 10 min. Finally, the slides were mounted and directly observed with an OLYMPUS BX63F fluorescence microscope. Meanwhile, the cell uptake was quantitatively analyzed by Novocyte Quanteon flow cytometry (ACEA Bioscience Inc., United States). The cells were collected, and washed with cold PBS three times. Finally, they were analyzed by flow cytometry.

To investigate the competitive inhibition of DOG/MOFA/ICG nanoparticle uptake via folate receptor-mediated endocytosis, the A549 cells were first incubated with free folate (1 mM) to block folate receptor binding and then further treated with DOG/MOFA/ICG nanoparticles for 4 h, then both attached and floating cells were collected, washed with cold PBS three times. Finally, they were analyzed by flow cytometry.

### 2.14 *In vitro* cytotoxicity study

A549 cells (1 × 10^4^ cells/100 µL/well) were incubated in a 96-well plate 24 h prior to the experiments, and the different concentrations of GEM, free DOG, ICG, and different nanoparticles were given to the cell. In order to determine the synergistic chemo-photothermal therapy effect, A549 cells were given without and with near-infrared laser irradiation (808 nm, 1 W/cm^2^) for 5 min after 24 h of drug administration. After being incubated for another 24 h, cells were washed twice with PBS, added fresh culture medium (100 μL), along with 10 μL of CCK-8 solution, and further incubated for 4 h in the dark. The absorbance was read at 450 nm with a VICTOR Nivo multimode plate reader. The following formula 4 was used to calculate cell viability. To estimate the additivity, synergy, or antagonism of combination treatments, the method of Chou and Talalay (Yang et al., 2021) was used to calculate the combination index (CI) values. The CI values were calculated as formula 5:

$$Cell\;viability\;\left(\%\right)=\frac{OD450\left(\mathrm{sample}\right)\;–\;OD450\left(\mathrm{blank}\right)}{OD450\left(\mathrm{control}\right)\;–\;OD450\left(\mathrm{blank}\right)}\times 100$$ (4)

$$CI=\frac{D_{1}}{{\left(D_{m}\right)}_{1}}+\frac{D_{2}}{{\left(D_{m}\right)}_{2}}+\frac{D_{1}D_{2}}{{\left(D_{m}\right)}_{1}{\left(D_{m}\right)}_{2}}$$ (5)

(*D*_m_)_1_ and (*D*_m_)_2_ represent the IC_50_ values of treatments 1 and 2 applied separately, while *D*_1_ and *D*_2_ are the IC_50_ values of treatments 1 and 2 applied as a combination.

### 2.15 Apoptosis analysis with flow cytometry

A549 cells were seeded in 6-well plates at 3.0 × 10^6^ cells per well in 1 mL of DMEM and cultured for 24 h. Then they were treated with GEM, free DOG, DOG nanoparticles, DOG/MOFA nanoparticles, DOG/ICG nanoparticles, and DOG/MOFA/ICG nanoparticles at GEM doses (8 μM) for 48 h. A549 cells without the same treatment were used as a control. To measure the cell apoptosis quantitatively, floating and attached cells were also collected, washed with ice-cold PBS three times, and stained with Annexin V-FITC and propodium iodide (PI). Finally, they were analyzed by flow cytometry.

### 2.16 *In vivo* pharmacokinetics

All *in vivo* studies were approved by the Institutional Animal Care and Use Committee (no. 2023-011) at Chengdu Medical College. The mice were randomly divided into two different groups for pharmacokinetic investigations. Each of them received a single intravenous injection of 10 mg/kg DOG/MOFA/ICG nanoparticles or 2.84 mg/kg free GEM (the free GEM administered concentration was consistent with that contained in the DOG/MOFA/ICG nanoparticles). At predetermined time intervals (0.083, 0.5, 1, 2, 4, 8, 12, and 24 h), the blood samples were collected, which were precipitated and purified with acetonitrile. The content of GEM or DOG in all samples was measured using a UFLC-MS/MS method. 1 μL sample was injected into an ultrafast liquid chromatography system (auto-sampler: SIL-30AC, chromatograph: LC-30AD, communications bus module: CBM-20A, prominence column oven: CTO-20AC, Shimadzu, Japan), equipped with Acquity UPLC BEH C18 column (100 mm × 2.1 mm, 1.7 mm; Waters). The concentrations were analyzed by AB SCIEX Qtrap 5500 mass spectrometer, with electrospray positive mode. The reported concentrations were mean ± SD values from 5 mice/time point/group, and DAS 3.0 software was used to fit pharmacokinetic parameters.

### 2.17 Statistical analysis

Statistical analyses were performed using GraphPad Prism 8.0 (GraphPad Software, San Diego, CA, United States). All data were analyzed by Student’s t-test and one-way analysis of variance (ANOVA). All data are expressed as mean ± standard deviation and obtained from at least three independent experiments. *p* < 0.05 was considered to indicate a statistically significant difference.

## 3 Results

### 3.1 Synthesis of amphiphilic GEM prodrugs and MOFA

#### 3.1.1 3′,5′-dioleoyl gemcitabine (DOG, #1)

White solid (yield 76.4%). ^1^H NMR (400 MHz, CDCl_3_), *δ* 7.77 (d, *J* = 8 Hz, 1 H), 7.51 (d, *J* = 8.0 Hz, 1 H), 6.45 (t, *J* = 8.0 Hz, 1 H), 5.46–5.21 (m, 5 H), 4.58–4.12 (m, 3 H), 2.56–2.30 (m, 4 H), 2.16–1.90 (m, 8 H), 1.76–1.53 (m, 4 H), 1.32–1.24 (m, 40 H), 0.88–0.86 (m, 6 H) (Supplementary Figure S1). HRMS (ESI/TOF-Q): calcd. for [C_45_H_75_F_2_N_3_O_6_+H^+^] 792.5697; found 792.5702 (Supplementary Figure S2).

#### 3.1.2 3′,5′-dilinoleoyl gemcitabine (DLG, #2)

White solid (yield 54.8%). ^1^H NMR (400 MHz, CDCl_3_) *δ* 8.95 (d, *J* = 8.6 Hz, 1 H), 7.81 (t, *J* = 8.0 Hz, 1 H), 6.21 (d, *J* = 8.6 Hz, 1 H), 5.68–5.49 (m, 4 H), 5.45–5.22 (m, 6 H), 4.57–4.30 (m, 2 H), 2.84–2.76 (m, 4 H), 2.39–2.22 (m, 4 H), 2.08–1.95 (m, 8 H), 1.74–1.57 (m, 4 H), 1.39–1.20 (m, 28 H), 0.94–0.83 (m, 6 H) (Supplementary Figure S3). HRMS (ESI/TOF-Q): calcd. for [C_45_H_71_F_2_N_3_O_6_+H^+^] 788.5384; found 788.5390 (Supplementary Figure S4).

#### 3.1.3 3′,5′-dimyristoyl gemcitabine (DMG, #3)

White solid (yield 68.3%). ^1^H NMR (400 MHz, CDCl_3_) *δ* 8.92 (d, *J* = 8.6 Hz, 1 H), 7.76 (t, *J* = 8.0 Hz, 1 H), 6.18 (d, *J* = 8.6 Hz, 1 H), 5.72–5.45 (m, 2 H), 4.90–4.68 (m, 2 H), 2.52–2.26 (m, 4 H), 1.76–1.56 (m, 4 H), 1.42–1.18 (m, 40 H), 0.95–0.83 (m, 5 H) **(** Supplementary Figure S5 **)**. HRMS (ESI/TOF-Q): calcd. for [C_37_H_63_F_2_N_3_O_6_+H^+^] 684.4758; found 684.4760 **(** Supplementary Figure S6 **)**.

#### 3.1.4 3′,5′-didecanoyl gemcitabine (DDG, #4)

White solid (yield 45.1%). ^1^H NMR (400 MHz, CDCl_3_) *δ* 8.92 (d, *J* = 8.5 Hz, 1 H), 7.76 (t, *J* = 8.0 Hz, 1 H), 6.20 (d, *J* = 8.5 Hz, 1 H), 5.40–5.07 (m, 2 H), 4.56–4.25 (m, 2 H), 2.45–2.24 (m, 4 H), 1.76–1.54 (m, 4 H), 1.33–1.15 (m, 24 H), 0.94–0.84 (m, 6 H) (Supplementary Figure S7). HRMS (ESI/TOF-Q): calcd. for [C_29_H_47_F_2_N_3_O_6_+H^+^] 572.3506; found 572.3500 (Supplementary Figure S8).

#### 3.1.5 3′,5′-diheptanoyl gemcitabine (DHG, #5)

White solid (yield 48.8%). ^1^H NMR (400 MHz, CDCl_3_) *δ* 8.93 (d, *J* = 8.5 Hz, 1 H), 7.02 (t, *J* = 8.0 Hz, 1 H), 6.18 (d, *J* = 8.5 Hz, 1H), 5.44–5.23 (m, 2 H), 4.70–4.54 (m, 2 H), 2.48–2.28 (m, 4 H), 1.71–1.62 (m, 4 H), 1.35–1.25 (m, 12 H), 0.95–0.82 (m, 6 H) **(** Supplementary Figure S9 **)**. HRMS (ESI/TOF-Q): calcd. for [C_23_H_35_F_2_N_3_O_6_+H^+^] 488.2567; found 488.2571 **(** Supplementary Figure S10 **)**.

#### 3.1.6 Synthesis of γ-octadecyl folate (MOFA)

Orange-yellow powder (60.5%). ^1^H NMR (400 MHz, DMSO-*d*_6_) *δ* 10.25 (s, 1 H), 9.14 (s, 1 H), 8.87 (s, 1 H), 7.67–7.52 (m, 2 H), 6.89–6.80 (m, 2 H), 6.63 (s, 2 H), 6.28 (s, 1 H), 4.55 (t, *J* = 8.4 Hz, 1 H), 4.39 (s, 2 H), 4.15–4.13 (m, 2 H), 2.43–2.29 (m, 2 H), 2.25–2.09 (m, 2 H), 1.68–1.51 (m, 2 H), 1.48–1.37 (m, 2 H), 1.32–1.23 (m, 24 H), 0.94–0.80 (m, 3 H) (Supplementary Figure S11). HRMS (ESI/TOF-Q): calcd. for [C_37_H_55_N_7_O_6_+H^+^] 694.4287; found 694.4293 (Supplementary Figure S12).

### 3.2 Self-assembly ability of amphiphilic GEM prodrugs

Four solvents miscible with water (DMSO, DMF, MeOH, and EtOH) were screened to prepare prodrug self-assembled nanoparticles by reprecipitation method (Wang et al., 2017). As shown in Supplementary Table S1, except DMG, amphiphilic GEM prodrugs could self-assemble into nanoparticles in DMSO, but they failed to do that in DMF as well as EtOH, and aggregated immediately, which indicated that DMSO was the optimal solvent for the self-assembly of amphiphilic prodrugs. Except for the DLG, the other amphiphilic prodrugs could not self-assemble into nanoparticles in methanol, indicating that the different lipid moieties introduced to GEM would affect the self-assembly process, which was consistent with the literature (Coppens et al., 2021).

### 3.3 Characterization of self-assembled nanoparticles

**FIGURE 3:**
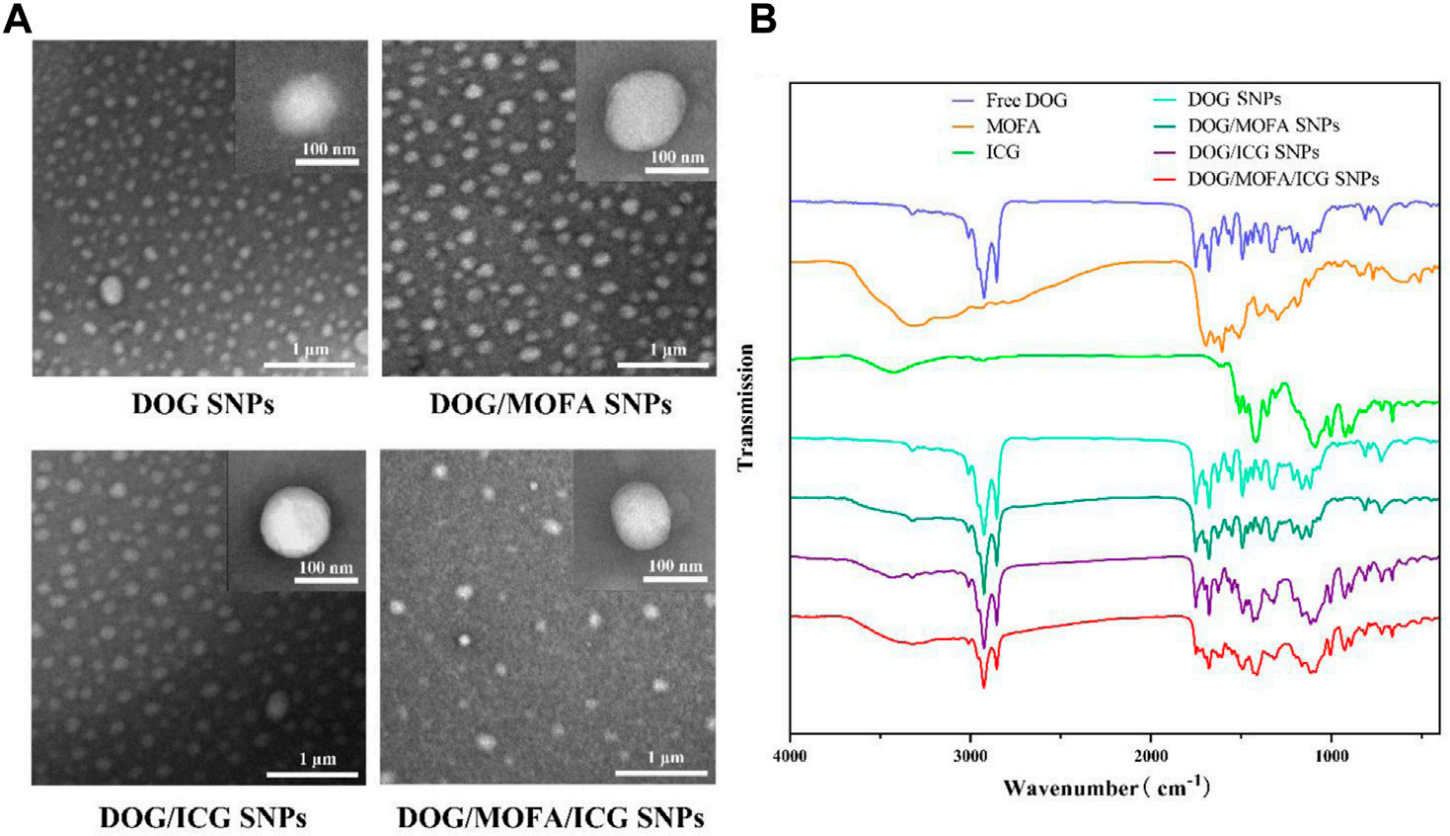
**(A)** The TEM images of different nanoparticles. **(B)** FT-IR spectra of different prodrugs and nanoparticles.

The average particle size of DOG, DOG/MOFA, DOG/ICG, and DOG/MOFA/ICG nanoparticles measured by the laser particle size analyzer were 94.74 ± 1.39 nm, 95.23 ± 0.89 nm, 102.6 ± 0.55 nm, and 109.2 ± 3.05 nm, respectively. The PDI of DOG, DOG/MOFA, DOG/ICG, and DOG/MOFA/ICG nanoparticles were 0.245 ± 0.010, 0.217 ± 0.006, 0.215 ± 0.011 and 0.188 ± 0.017, respectively (Table 1). The potential zeta of DOG, DOG/MOFA, DOG/ICG, and DOG/MOFA/ICG nanoparticles were −30.30 ± 1.16 mV, −24.00 ± 0.90 mV, −29.90 ± 0.93 mV and −30.10 ± 0.70 mV, respectively (Table 1). TEM showed that the shape of different nanoparticles was almost spherical (Figure 3A). Representative absorption peaks of MOFA and ICG were observed from the UV-Vis spectra of self-assembled nanoparticles and were slightly red-shifted (Supplementary Figure S13A). Then, the fluorescence properties of free ICG, DOG/ICG, and DOG/MOFA/ICG nanoparticles were examined. The fluorescence emission peak of ICG, ICG in DOG/ICG, and DOG/MOFA/ICG nanoparticles was around 814 nm (Supplementary Figure S13B).

**TABLE 1:** The size distribution, PDI, and zeta potential of different nanoparticles.

Formulations	Mean diameter (nm) *	PDI *	Zeta potential (mV) *
DOG SNPs	94.74 ± 1.39	0.245 ± 0.010	−30.30 ± 1.16
DOG/MOFA SNPs	95.23 ± 0.89	0.217 ± 0.006	−24.00 ± 0.90
DOG/ICG SNPs	102.6 ± 0.55	0.215 ± 0.011	−29.90 ± 0.93
DOG/MOFA/ICG SNPs	109.2 ± 3.05	0.188 ± 0.017	−30.10 ± 0.70

### 3.4 Assembly mechanism of DOG/MOFA/ICG nanoparticles

Assembly mechanisms of DOG/MOFA/ICG nanoparticles were explored by molecular dynamics simulation. It could be found that various intermolecular interactions were involved in the assembly process, such as hydrogen bonds, π-π stacking, π-cation, and halogen hydrophobic interaction (Supplementary Figure S14A). The results of molecular dynamics simulation also exhibited that DOG, MOFA, and ICG molecules aggregated into clusters (nanoparticles) in an aqueous solution within 100 ns (Supplementary Figure S14B). According to the ^1^H NMR spectrum (Supplementary Figure S14C and Supplementary Figure SS15–S20), the proton signal of ∼8.8 ppm attributed to the amino group of DOG was shifted to ∼7.9 ppm and ∼7.8 ppm in DOG/MOFA nanoparticles and DOG/MOFA/ICG nanoparticles respectively. Furthermore, the Fourier transform infrared (FT-IR) spectrum of different prodrugs and nanoparticles was also recorded. In different nanoparticles, the characteristic peaks of different chemical components appeared in the infrared spectrum, such as ν(C=N), ν(C=O) and ν(C-H) of DOG, ν(N-H) of MOFA, and ν(S=O) of ICG. Compared with ν(C=N) (∼1,627 cm^−1^, characteristic of the heterocycle) and ν(C=O) (∼1750 cm^−1^) signals of DOG, ν(N-H) (∼3,310 cm^−1^) signals of MOFA as well as ν(S=O) (∼1,088 cm^−1^, characteristic of the sulfonic acid group) of ICG, a low-wavenumber shift of ν(C=N) (∼1,610 cm^−1^), two high-wavenumber shifts of ν(S=O) (∼1,114 cm^−1^) and ν(N-H) (∼3,320 cm^−1^) with the weaken signal of ν(C=O) (∼1750 cm^−1^) could be observed in DOG/MOFA/ICG nanoparticles (Figure 3B and Supplementary Figure S21–S28).

### 3.5 Loading and drug release

Standard curves for DOG and ICG have been established (Supplementary Figure S29). The DL of DOG in DOG/ICG, DOG/MOFA, and DOG/MOFA/ICG nanoparticles were 92.01% ± 3.25%, 90.70% ± 6.84%, and 85.35% ± 4.61%, respectively. The EE of DOG in DOG/ICG, DOG/MOFA, and DOG/MOFA/ICG nanoparticles were 80.90% ± 4.52%, 82.60% ± 5.24%, and 92.65% ± 5.71%, respectively. The DL of the photothermal therapy moiety ICG in DOG/ICG and DOG/MOFA/ICG nanoparticles were 7.99% ± 0.89% and 6.84% ± 0.93%, respectively. The EE of the photothermal therapy moiety ICG in DOG/ICG and DOG/MOFA/ICG nanoparticles were 70.25% ± 2.18% and 74.26% ± 2.84%, respectively.

**FIGURE 4:**
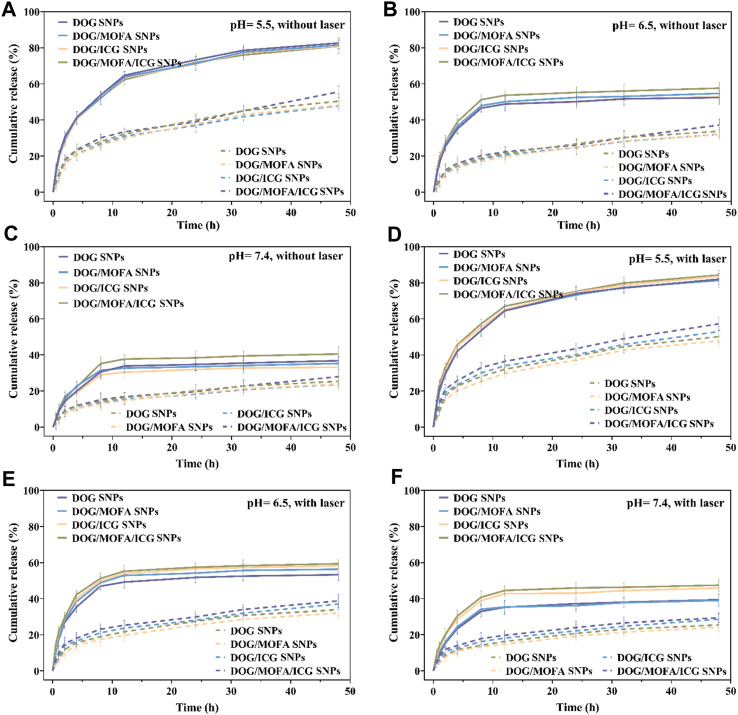
The GEM release *in vitro* of different nanoparticles without laser irradiation **(A–C)** or with laser irradiation **(D–F)** under different pH conditions with (solid line)/without (dashed line) esterase (30 U/mL). Data are presented as the mean ± standard deviation (*n* = 3).

The releasing profiles of GEM and ICG in different nanoparticles were studied via dialyzing method in PBS without/with esterase, without/with laser irradiation in different pH (37°C). As depicted in Figures 4A–F and Supplementary Figure S30, there was less than 42% GEM and ICG released from nanoparticles in a neutral PBS solution (pH 7.4) within 48 h. In contrast, more than 55% GEM and ICG in PBS (pH 6.5) and more than 82% GEM and ICG in PBS (pH 5.5) were released from nanoparticles. It was worth noting that there was less GEM release in the absence of esterase condition, suggesting that the GEM prodrug DOG was also released from the dialysis bag. Meanwhile, the laser irradiation could slightly boost GEM and ICG release (Figures 4D–F and Supplementary Figure S30), which was less obvious than acidic pH which was easier to break the ester bond. In addition, the drug release was significantly reduced in the absence of esterase, indicating that those nanoparticles were esterase-responsive release.

### 3.6 *In vitro* stability

It was found that the zeta potential and size of different nanoparticles in PBS (pH 7.4), 10% FBS solution, and DMEM under 4°C and 37°C almost remained unchanged within 7 days respectively (Supplementary Figure S31A–F). Moreover, these nanoparticles were stable under laser irradiation within three cycles, with a slight change in the size and PDI (Supplementary Figures S31G, H).

### 3.7 Determination of CMC

As shown in Supplementary Figure S32, the intersection of two lines was on behalf of the value of CMC. The CMC values of DOG, DOG/MOFA, DOG/ICG, and DOG/MOFA/ICG nanoparticles were calculated to be 0.00038, 0.00048, 0.00050, and 0.00055 μM, respectively.

### 3.8 *In vitro* photothermal effect

**FIGURE 5:**
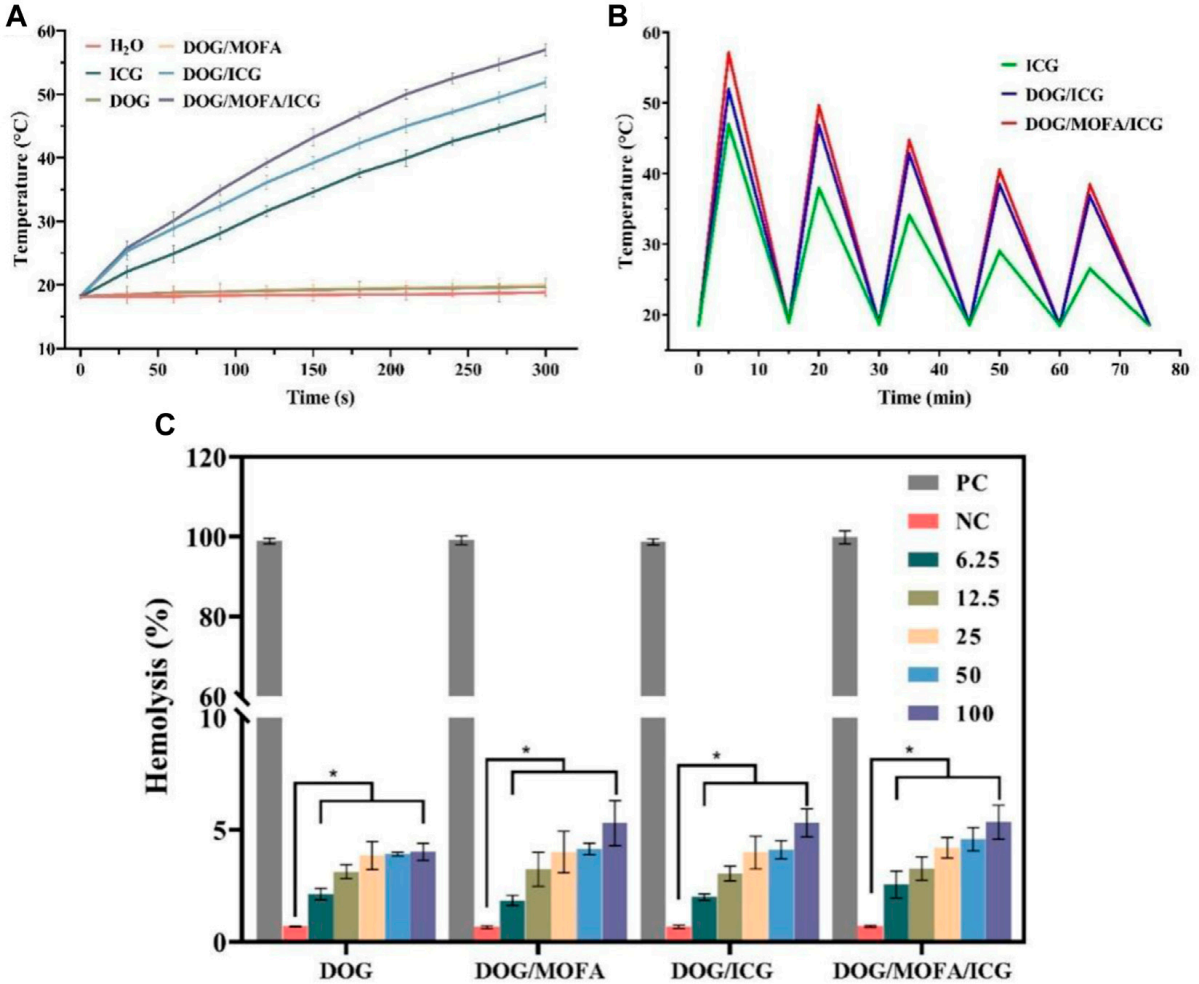
**(A)** Photothermal heating curves of water, free ICG, and different nanoparticles under 808 nm laser irradiation (1 W/cm^2^, 5 min) and **(B)** under 808 nm laser irradiation (1 W/cm^2^) during five natural cooling cycles. **(C)** Hemolysis assay with different concentrations (μg/mL) of different nanoparticles. PC: positive control. NC: negative control. Data are presented as the mean ± standard deviation (*n* = 3), statistical significance ^*^ is different concentration group compared with the negative control group: ^*^ *p* < 0.05.

The temperature change of the nanoparticle solution under 808 nm laser irradiation was used for evaluating the photothermal effect of nanoparticles. After near-infrared laser irradiation (1 W/cm^2^) for 5 min, the temperature of water, DOG, and DOG/MOFA nanoparticles hardly increased. The temperature of free ICG reached about 50°C, while the temperatures of DOG/ICG and DOG/MOFA/ICG nanoparticles reached 51.9°C and 57.0°C, respectively (Figure 5A). Besides, the photothermal stability of DOG/ICG and DOG/MOFA/ICG nanoparticles was investigated also. A heat generation-loss profile was obtained by recording the change in temperature throughout four ON/OFF cycles (Figure 5B). After four ON/OFF cycles of laser irradiation, the maximum temperature of DOG/ICG and DOG/MOFA/ICG nanoparticles could still reach about 40°C.

### 3.9 Hemolysis assay

According to the results of the hemolysis assay, only insignificant hemolysis (approximately 5%) was observed, which shows that these different nanoparticles had good biocompatibility for *in vivo* profiles (Figure 5C and Supplementary Figure S32).

### 3.10 Cellular uptake

**FIGURE 6:**
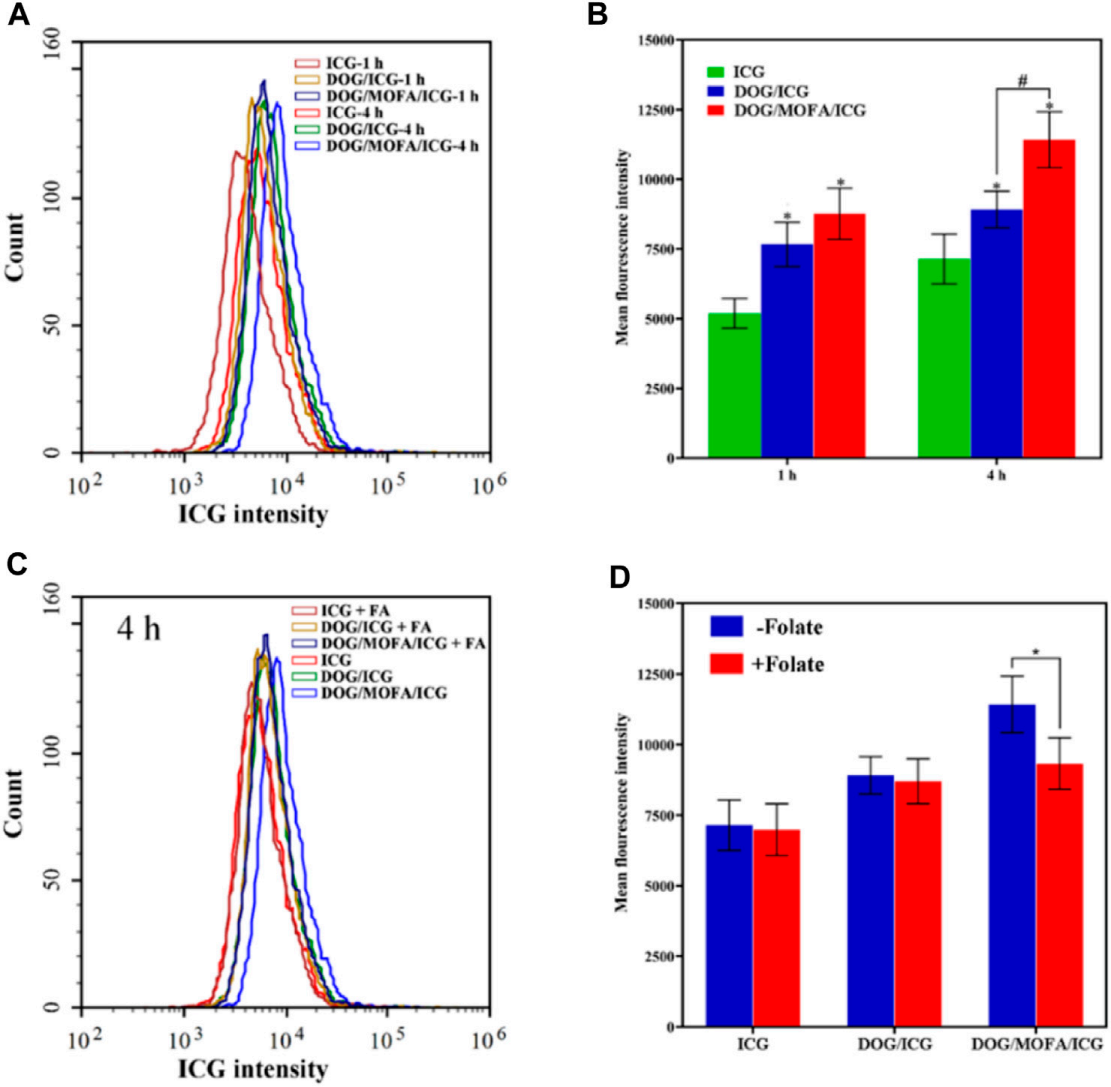
The cellular ICG fluorescence intensity of DOG/ICG and DOG/MOFA/ICG nanoparticles at different incubation time intervals (1 h, 4 h) in A549 cells **(A)** and the quantitative results were analyzed by flow cytometry **(B)**. Blocking experiment after pretreatment with excess folate **(C)** and the quantitative results were analyzed by flow cytometry **(D)**. Data are presented as the mean ± standard deviation (*n* = 3), statistical significance ^*^ is compared with the ICG group, and ^#^ is compared with the DOG/ICG group: ^*^ and ^#^ *p* < 0.05, Scale bar: 20 μm.

ICG-encapsulated DOG/ICG and DOG/MOFA/ICG nanoparticles enable cell imaging due to the autofluorescence of ICG, which was used to qualitatively analyze the cellular uptake of DOG/ICG and DOG/MOFA/ICG nanoparticles by fluorescence microscope. The free ICG was used as a control. As displayed in Figures 6A, B and Supplementary Figure S33, the mean ICG fluorescence intensity of DOG/ICG and DOG/MOFA/ICG nanoparticles was stronger than that of the free ICG group, and the average ICG fluorescence intensity of DOG/MOFA/ICG nanoparticles was stronger than that of DOG/ICG nanoparticles. Moreover, with prolonged co-incubation time of nanoparticles and A549 cells, the increased fluorescence signal indicated that cell uptake of DOG/ICG and DOG/MOFA/ICG nanoparticles was time-dependent. In addition, the fluorescence intensity of DOG/MOFA/ICG nanoparticles + free folate (pre-treatment with 1 mmol/L) was considerably lower than that of DOG/MOFA/ICG nanoparticles in the competitive inhibition experiment (Figures 6C, D), suggesting that the high uptake behavior of DOG/MOFA/ICG nanoparticles may be attributed to folate receptor-mediated active transport (active targeting).

### 3.11 *In vitro* cytotoxicity

**FIGURE 7:**
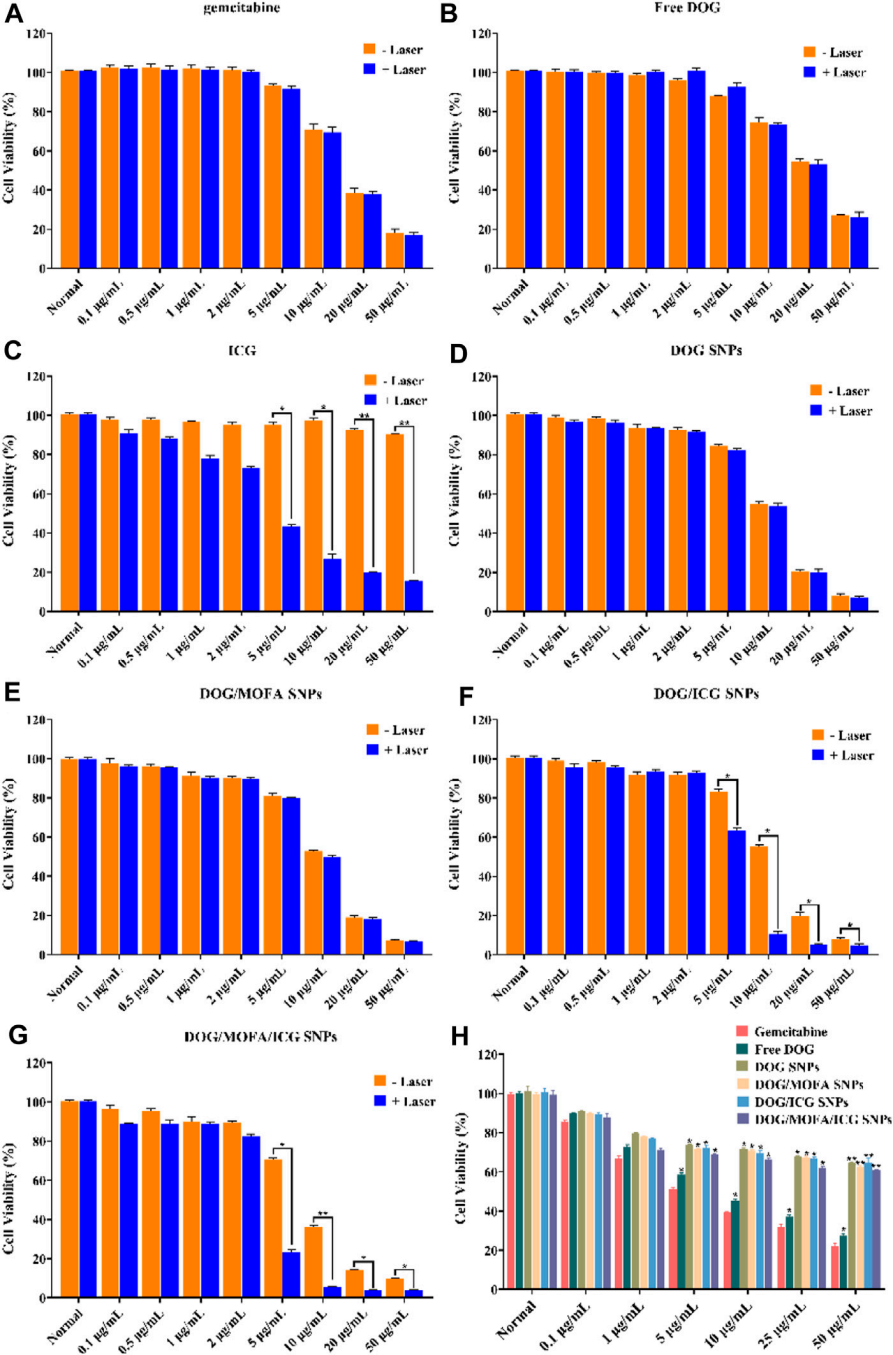
Cell viability of CCK-8 assay. **(A–G)** Cell viability of A549 cells incubated with different concentrations of GEM, ICG, free DOG, and different nanoparticles for 48 h with/without laser irradiation (808 nm, 1 W/cm^2^). **(H)** Cell viability of L929 cells incubated with different concentrations of GEM, ICG, free DOG, and different nanoparticles. Data are represented as the mean ± standard deviation (*n* = 3), statistical significance ^*^ is compared with the GEM group, and ^^^ is compared with the normal group: ^*^ *p* < 0.05, ^**^ *p* < 0.01.

*In vitro* cytotoxicity of self-assembled nanoparticles against A549 cells was assessed by CCK-8 assay. The cell viability of A549 cells decreased with increasing drug concentrations in different self-assembled nanoparticles, and laser irradiation caused a further decrease in their viability (Figure 7A–G). The IC_50_ values and dose-response curves of drugs and different nanoparticles are listed in Table 2 and Supplementary Figure S33. Additionally, the relationship between dose and combination index (CI) in DOG/ICG and DOG/MOFA/ICG nanoparticles was summarized in Table 3.

**TABLE 2:** IC_50_ values of different formulations against A549 cells incubated with different concentrations of GEM, ICG, free DOG, and different nanoparticles for 48 h.

Formulations	IC_50_ (μg/mL) without laser irradiation	IC_50_ (μg/mL) with laser irradiation
GEM	13.08 ± 0.43	13.00 ± 0.53
Free DOG	23.33 ± 2.52^**^ ^a^	22.56 ± 0.89^**^ ^b^
ICG	–	3.792 ± 0.07^**^ ^b^
DOG	10.62 ± 0.02^*^ ^a^	10.61 ± 0.45^*^ ^b^
DOG/MOFA	10.44 ± 0.11^*^ ^a^	10.06 ± 0.07^*^ ^b^
DOG/ICG	10.65 ± 0.22^*^ ^a^	5.71 ± 0.13^**^ ^b^
DOG/MOFA/ICG	7.70 ± 0.04^**^ ^a^	3.69 ± 0.05^**^ ^b^

Note: Data is represented as the mean ± standard deviation (*n* = 3). Statistical significance.

a Is compared with the GEM, without laser irradiation group.

b Is compared with the GEM, with laser irradiation.

* *p*< 0.05, ** *p*< 0.01.

**TABLE 3:** The relationship between dose and combination index (CI) in DOG/ICG and DOG/MOFA/ICG nanoparticles.

DOG/ICG				DOG/MOFA/ICG			
Dose DOG	Dose ICG	Effect	CI	Dose DOG	Dose ICG	Effect	CI
0.1	0.009	1.000	11.156	0.1	0.008	1.000	10.554
0.2	0.017	0.956	0.230	0.2	0.016	0.891	0.114
0.5	0.043	0.958	0.600	0.5	0.040	0.891	0.283
1.0	0.087	0.935	0.859	1.0	0.080	0.887	0.550
2.0	0.174	0.930	1.620	2.0	0.160	0.823	0.762
5.0	0.434	0.635	0.964	5.0	0.401	0.234	0.283
10.0	0.868	0.106	0.296	10.0	0.801	0.054	0.177
20.0	1.737	0.052	0.346	20.0	1.603	0.039	0.283
50.0	4.342	0.050	0.839	50.0	4.007	0.038	0.694

Note: CI < 0.1, very strong synergism; CI, 0.1–0.3, strong synergism; CI, 0.3–0.7, synergism; CI, 0.7–0.85, moderate synergism; CI, 0.85–0.90, light synergism; CI, 0.90–1.10, nearly additive; CI, 1.10–1.20, slight antagonism; CI, 1.20–1.45, moderate antagonism; CI, 1.45–3.3, antagonism; CI, 3.3–10, strong antagonism; and, CI > 10, very strong antagonism.

Next, DOG/MOFA/ICG nanoparticles were investigated for the capability of targeted delivery to get selective inhibition of A549 cancer cells over the L929 normal cells. The DOG/MOFA and DOG/MOFA/ICG nanoparticles were much more cytotoxic to A549 cells than DOG nanoparticles at all doses (Figure 7A–G). Instead, incubating the above-mentioned drugs and nanoparticles with L929 cells, similar cytotoxicity of different nanoparticles to L929 cells could be observed (Figure 7H), indicating the selectivity of these nanoparticles to tumor cells.

### 3.12 Apoptosis analysis with flow cytometry

**FIGURE 8:**
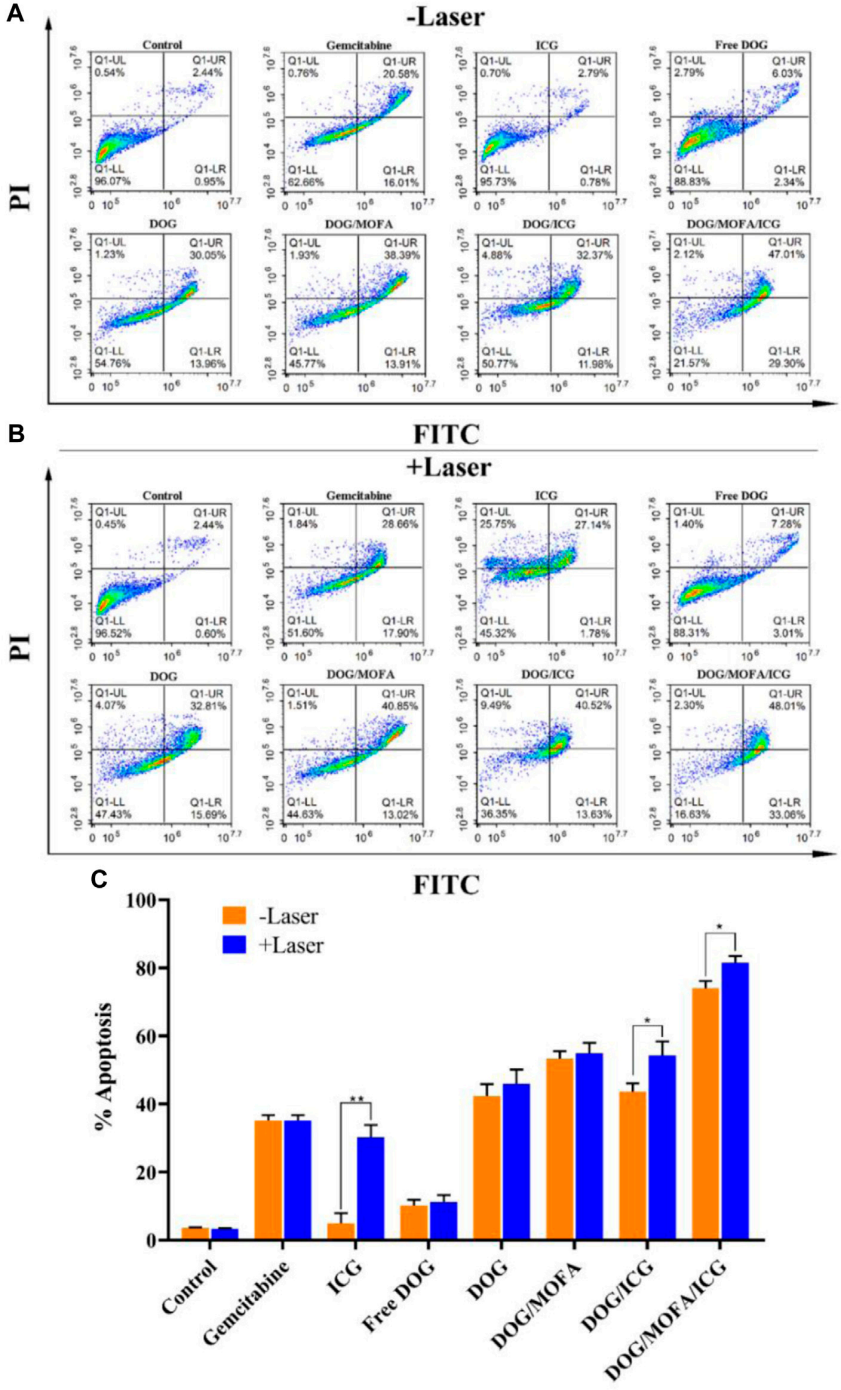
Flow cytometry analysis for A549 cells apoptosis induced by GEM, ICG, free DOG, and different nanoparticles without **(A)** or with laser irradiation **(B)**. The upper right quadrant (Annexin V-FITC^+^/PI^+^) represents the late apoptotic stage cells and the lower right quadrant (Annexin V-FITC^+^/PI^−^) represents the early apoptotic stage cells. The total proportion of apoptotic cells (early and late) in the **(C)** A549 cell. Data are expressed as the mean ± standard deviation (*n* = 3). *p < 0.5 and **p < 0.01.

To confirm the ability of different nanoparticles to induce apoptosis, flow cytometry analysis was used for quantification. As illustrated in Figure 8A, the percentages of living cells for the control group and the ICG without laser group were 96% and 95% respectively, during the treatment period, which showed the high safety of ICG. Compared with GEM group, the total proportion of apoptotic cells in the free DOG (amphiphilic prodrug) group was obviously decreased, indicating prodrug could reduce cytotoxicity in a short period of time. The different nanoparticles group showed higher total apoptosis rates compared with the GEM group, which indicated nanoparticles could indeed significantly increase the cytotoxicity to A549 cells. What’s more, the introduction of targeting moieties promoted the antitumor effects of nanoparticles.

As illustrated in Figure 8B, the effects of laser irradiation on cytotoxicity were not significant in all groups without ICG, which indicated short-term laser irradiation was basically non-toxic to cells. In addition, laser irradiation significantly aggravated apoptosis in groups containing ICG, and it was found that DOG/MOFA/ICG nanoparticles induced significant apoptosis (81.07%) under 808 nm laser irradiation (1 W/cm^2^, 5 min). The results demonstrated DOG/MOFA/ICG nanoparticles possessed excellent antitumor activity via synergetic chemo-photothermal therapy.

### 3.13 *In vivo* pharmacokinetics

**FIGURE 9:**
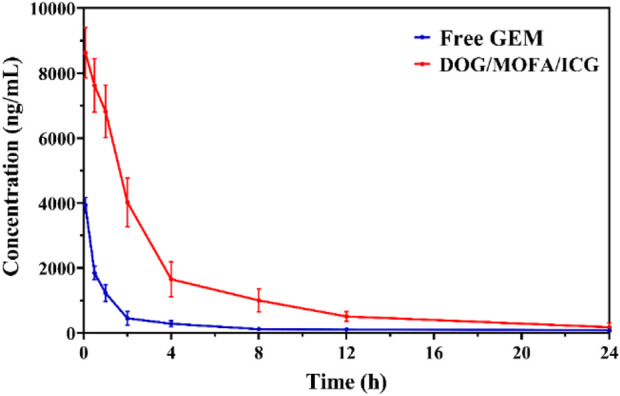
The free GEM and DOG concentrations in plasma after a single intravenous administration of the free GEM and DOG/MOFA/ICG nanoparticles at 0–24 h time points were measured by LC-MS/MS.

To explore whether DOG/MOFA/ICG nanoparticles could ameliorate the pharmacokinetics properties of free GEM, the pharmacokinetic studies of free GEM and DOG/MOFA/ICG nanoparticles were conducted. The mice were given the free GEM or DOG/MOFA/ICG nanoparticles intravenously at an equivalent dose of 2.84 mg/kg GEM. At multiple time points, the plasma concentrations of free GEM and DOG were measured. As found in Table 4 and Figure 9, the half-life (t_1/2_) of DOG in plasma was 5.66 ± 0.81 h, which was significantly longer than that of the free GEM group (3.08 ± 0.68 h). Furthermore, at 24 h after DOG/MOFA/ICG nanoparticles treatment, it was found that the plasma concentrations of DOG were higher than that of GEM, which indicated that DOG/MOFA/ICG nanoparticles administrated intravenously could have a sustained-release effect to maintain a long-term anti-tumor activity.

**TABLE 4:** Pharmacokinetic parameters of the free GEM and DOG/MOFA/ICG nanoparticles after a single intravenous administration.

Parameters	Free GEM	DOG/MOFA/ICG nanoparticles
t_1/2z_ (h)	3.08 ± 0.68	5.66 ± 0.81^*^
AUC_0-∞_ (ng/L*h)	5979.91 ± 421.25	30,602.85 ± 1810.61^*^
MRT_0-∞_ (h)	4.93 ± 0.43	5.62 ± 0.53
CL_z_ (L/h/kg)	4.47 ± 0.59	3.26 ± 0.32^*^

Note: ^*^ *p* < 0.05 by Student’s t-test between free GEM and DOG/MOFA/ICG nanoparticles administration groups.

## 4 Discussion

The carrier-free chemo-photothermal nanoparticle therapy has become a promising tactic to enhance anti-cancer therapeutic efficacy owing to the combination of chemotherapy and photothermal therapy, with improved chemotherapy drug pharmacodynamics and pharmacokinetics, high drug loading, and reduced toxicity. GEM, as a typical clinical first-line anti-tumor nucleoside analog, the major shortcomings were short half-life in plasma, rapid inactivation by the enzyme, and susceptibility to drug resistance. To overcome its deficiencies in the application, GEM prodrug was synthesized, and established the self-assembly of GEM nanoparticles to obstruct medication release. Recently, fatty acids were introduced to the amino group of GEM to prepare amphiphilic prodrugs for self-assembly nanoparticles (Coppens et al., 2021), but the protocol was complicated and time-consuming. Thus, we decided to introduce the fatty acids onto the sugar moiety of GEM instead of the amino group to obtain amphiphilic prodrugs conveniently. The synthesis of amphiphilic prodrugs was shown in Figure 2 and the chemical structure of GEM amphiphilic prodrugs was identified by ^1^H NMR spectroscopy and TOF-Q mass spectroscopy. From the integral ratio of the methine protons (*δ* 7.79 and 7.53 ppm) in the pyrimidine ring and methyl protons (*δ* 0.90 ppm) in fatty acid, it could be proved that two oleic acid chains were coupled to the GEM successfully. Comparing the ^1^H NMR spectrum of free GEM and amphiphilic prodrugs, it was found that the signs of 3′-OH (*δ* 6.24 ppm) and 5′-OH (*δ* 5.20 ppm) disappeared, the chemical shifts of 3′-H and 5′-H of sugar moiety shifted to 5.39 and 4.43 ppm from 4.14 ppm as well as 3.73 and 3.63 ppm respectively, which verified that two oleic acid chains were coupled to 3′-OH and 5′-OH of sugar moiety (Supplementary Figures S1, S3, S5, S7, S9). Also, the structures of DOG, DLG, DMG, DDG, and DHG were confirmed by molecular weights measured by TOF-Q mass spectroscopy of 792.5702, 788.5390, 684.4760, 572.3500, and 488.2571, respectively (Supplementary Figures S2, S4, S6, S8, S10). The folate receptor is commonly overexpressed on various tumor types and frequently used for active targeted nanosystems delivery. Here, 1-octadecanol was introduced to folate to synthesize amphiphilic MOFA, which was identified by the integral ratio of the methine protons (δ 9.14 ppm) in N-heterocycle and methyl protons (δ 0.89 ppm) in 1-octadecanol (Supplementary Figure S11), as well as a molecular weight of 694.4293 by TOF-Q mass spectroscopy (Supplementary Figure S12).

The size of nanosystems ranging from 70 to 200 nm allows for more accumulation in tumors (Jeevanandam et al., 2016). To obtain the suitable size of co-self-assembly carrier-free GEM nanoparticle loading indocyanine green (DOG/MOFA/ICG) for an actively targeted combination of chemotherapy and photothermal therapy, four solvents miscible with water (DMSO, DMF, MeOH, and EtOH) were screened by reprecipitation method. It was found that DMSO was the optimal solvent for the self-assembly of amphiphilic prodrugs. Among all prodrugs, the nanoparticle size formed by DOG was the smallest (∼94.74 nm), with the highest value of Zeta potential (−30.30 mV) and mono-dispersity, indicating that the DOG could form the most stable nanoparticle (Supplementary Table S1). The average particle size of DOG/MOFA/ICG nanoparticles obtained by the laser particle size analyzer was 109.2 ± 3.05 nm. The PDI of the DOG/MOFA/ICG nanoparticle was 0.188 ± 0.017, with a potential zeta of −30.10 ± 0.70 mV. It was observed that the zeta potential and size of nanoparticles in PBS (pH 7.4), 10% FBS solution, and DMEM under 4°C and 37°C almost remained unchanged within 7 days respectively (Supplementary Figure S31). Moreover, these nanoparticles were stable under laser irradiation within three cycles, with slight changes in the size, PDI, and zeta (Supplementary Figures S31G–I). The self-assembly nanoparticles could be kept in reserve for a long time, with excellent stability. The molecular dynamics (MD) calculations showed that the excellent stability of the nanoparticles originated from the interactions between folate prodrug MOFA and DOG, such as hydrogen bond, π-π stacking, π-cation and halogen hydrophobic interaction **(** Supplementary Figure S13 **)**, which was verified by ^1^H-NMR and infrared spectra (Supplementary Figure S14–S28). The pteridine group of folate was similar to guanine, which can form hydrogen bonds, and π−π stacking with GEM prodrug DOG, which was similar to guanine and cytosine base pairing. Furtherly, the hydrophobic chain tails of folate prodrug MOFA and GEM prodrug DOG could interact with each other by hydrophobic action. The interactions between different moieties improved the structural integrity of the self-assembled nanoparticles’ stability.

To better understand the characteristics of co-self-assembly carrier-free nanoparticles, the anti-blood dilution properties and releasing profiles of different nanoparticles were investigated. All self-assembled nanoparticles had a rather low CMC value; suggesting these self-assembled nanoparticles were stable to anti-dilution during blood circulation. The releasing profiles showed that the different nanoparticles could have pH/photo and enzyme-responsive drug release properties because of the break of the ester bond. The release behavior of ICG was generally consistent with GEM (Figure 4 and Supplementary Figure S30), which proved that ICG was successfully encapsulated inside the nanoparticles to ensure the synergism of chemotherapy and photothermal therapy. *In vivo* pharmacokinetic studies showed that DOG/MOFA/ICG nanoparticles displayed a pharmacokinetic profile dramatically different from that of free GEM. Compared to the free GEM, the plasma concentration of DOG after the treatment of DOG/MOFA/ICG nanoparticle declined slower than that after the treatment of free GEM over time, and the t_1/2_ and AUC_0-∞_ of the DOG nanoparticle increased 1.84, 5.12 fold, respectively (Table 4). The self-assembly of DOG/MOFA/ICG nanoparticles did help prolong their circulation time in the blood and enhance bioavailability.

The photothermal effect of nanoparticles was evaluated. After near-infrared laser irradiation (1 W/cm^2^) for 5 min, the temperature of free ICG reached about 50°C, while the temperatures of DOG/ICG and DOG/MOFA/ICG nanoparticles reached 51.9°C and 57.0°C respectively, which suggested the photothermal efficiency of nanoparticles loading ICG was significantly improved (Figure 5A). The reasons might be that ICG-loaded self-assembled nanoparticles own a highly condensed concentration than free ICG, resulting in higher light-to-heat conversion efficiency and less heat loss. Even after four ON/OFF cycles of laser irradiation, the maximum temperature of DOG/ICG and DOG/MOFA/ICG nanoparticles could still reach about 40°C, indicating that DOG/ICG and DOG/MOFA/ICG nanoparticles exhibited high photothermal stability, which could result from the interaction between the ICG and its adjacent solvent was reduced. In addition, weak intermolecular interactions restricted the movement of ICG molecules within the nanoparticles to reduce photothermal dissipation.

It is important to evaluate the safety and efficacy of the nanoparticles. Only insignificant hemolysis (approximately 5%) was observed in the nanoparticles compared to the proportion of hemolysis in GEM (approximately 35%) (Jeswani et al., 2015), and the *in vitro* toxicity of the nanoparticles to L929 cells was significantly lower than that of GEM itself (Figure 7). These results indicated the obtained nanoparticles were highly biocompatible. Cell lethality was well known to correlate with the cellular uptake of nanodrugs. Cell uptake and competitive uptake experiments demonstrated that DOG/MOFA/ICG nanoparticles increased cellular uptake of nanoparticles through folate-mediated active targeting (Figure 6F). Thus DOG/MOFA/ICG nanoparticles were the most effective compared to other nanoparticles. The high efficiency of DOG/MOFA/ICG combined with laser irradiation *in vitro* might be owed to the synergistic effect between chemotherapy and photothermal therapy. The temperature increase induced by laser radiation could enhance the permeability of tumor cell membranes and increase the opportunities for GEM to act on the nucleus of the tumor cell. At the same time, GEM release triggered by laser radiation could enhance the effectiveness of chemotherapy. Therefore, chemo-photothermal nanoparticle therapy is a promising strategy for improving anti-cancer therapeutic efficacy. The CI could reflect the synergism of chemotherapy and photothermal therapy. As shown in Table 3, DOG and ICG synergistically inhibited tumour growth at almost all doses, except very low doses. The lever of synergy was related to the dosage. When the concentrations of DOG and ICG were 0.2 and 0.017 μg/mL, the value of CI was the smallest (0.23 for DOG/ICG and 0.114 for DOG/MOFA/ICG), indicating strong synergism. The results suggested that DOG and ICG had a good mutual promotion effect. In addition, flow cytometry analysis to quantify apoptosis induced by different nanoparticles was consistent with the antitumor effect of nanoparticles.

## 5 Conclusion

A new folate-receptor-targeted carrier-free GEM nanoparticle loading indocyanine green for chemo-photothermal combination therapy was developed by the co-self-assembly between chemotherapeutic prodrug, active targeting agent, and photothermal agent. The readily available and highly reproducible DOG/MOFA/ICG nanoparticles showed monodispersity, high drug payload, excellent stability in physiological solutions, high photothermal conversion efficacy, the pH-/photo-responsive release of the drug in solution, sustained release activity, and higher bioavailability compared with free GEM *in vivo* as well as preferential cell uptake and cytotoxicity due to the active targeting of folate. This co-self-assembly of therapeutic, targeting, and photothermal agents is able to be used for the design of highly simplified and efficient integrated nanosystems for active targeting cancer synergistic therapy.

## Data Availability

The original contributions presented in the study are included in the article/Supplementary Material, further inquiries can be directed to the corresponding author.
