# Supplementary material for: Folate-receptor-targeted co-self-assembly carrier-free gemcitabine nanoparticles loading indocyanine green for chemo-photothermal therapy
Source: Front Bioeng Biotechnol. 2023 Sep 21;11:1266652. doi: 10.3389/fbioe.2023.1266652 (PMC10557076; doi:10.3389/fbioe.2023.1266652)
Supplement: Supplementary file 1 [file DataSheet1.docx]

Supplementary Material

**Table S1.** Formulation conditions for the GEM prodrug and resulting nanoparticle characterization.

|  | **prodrug** | **organic solvent** | **Mean diameter (nm)** * | **PDI** * | **Zeta potential (mV)** * |
| --- | --- | --- | --- | --- | --- |
| #01 | DOG | DMSO | 94.74 ± 1.4 | 0.245 ± 0.01 | -30.3 ± 1.2 |
|  |  | DMF | aggregation | | |
|  |  | MeOH | aggregation | | |
|  |  | EtOH | aggregation | | |
| #02 | DLG | DMSO | 174.0 ± 6.5 | 0.315 ± 0.15 | -28.4 ± 2.5 |
|  |  | DMF | aggregation | | |
|  |  | MeOH | 207.4 ± 8.4 | 0.307 ± 0.09 | -27.5 ± 1.9 |
|  |  | EtOH | aggregation | | |
| #03 | DMG | DMSO | aggregation | | |
|  |  | DMF | aggregation | | |
|  |  | MeOH | aggregation | | |
|  |  | EtOH | aggregation | | |
| #04 | DDG | DMSO | 138.6 ± 4.5 | 0.168 ±0.05 | -28.3 ± 1.9 |
|  |  | DMF | aggregation | | |
|  |  | MeOH | aggregation | | |
|  |  | EtOH | aggregation | | |
| #05 | DHG | DMSO | 421.6 ± 34.4 | 0.338 ± 0.18 | -27.8 ± 3.5 |
|  |  | DMF | aggregation | | |
|  |  | MeOH | aggregation | | |
|  |  | EtOH | aggregation | | |

* Measured by dynamic light scattering (Mean ± SD, n= 3).


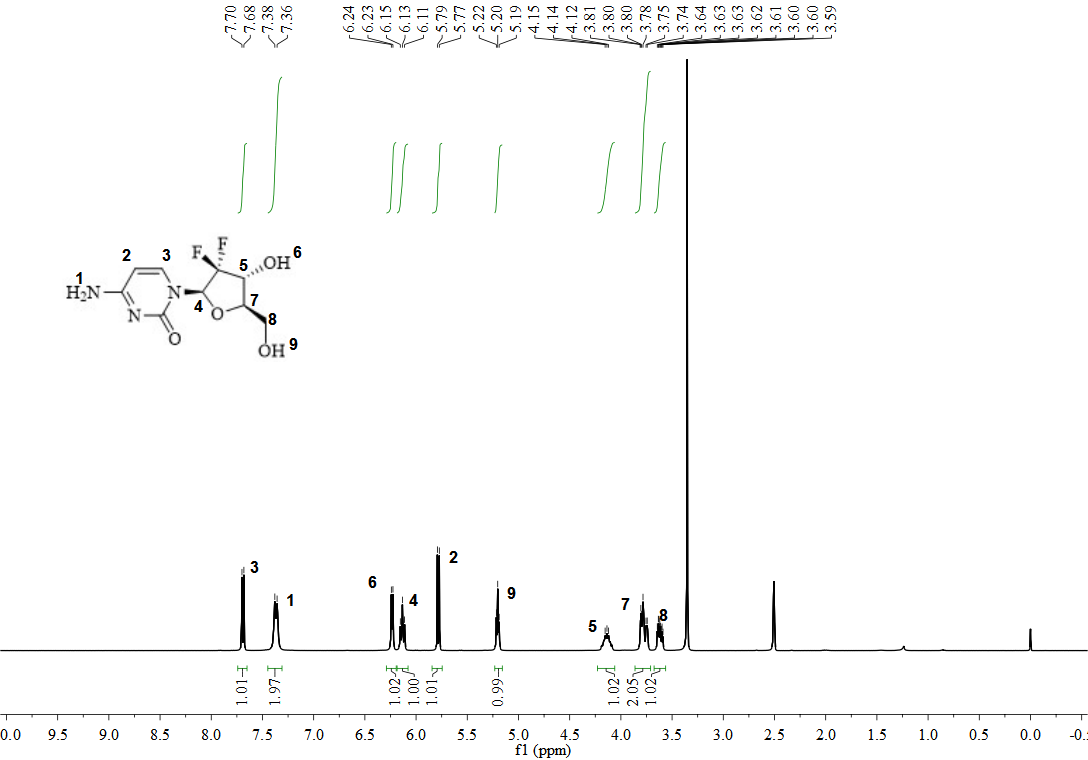

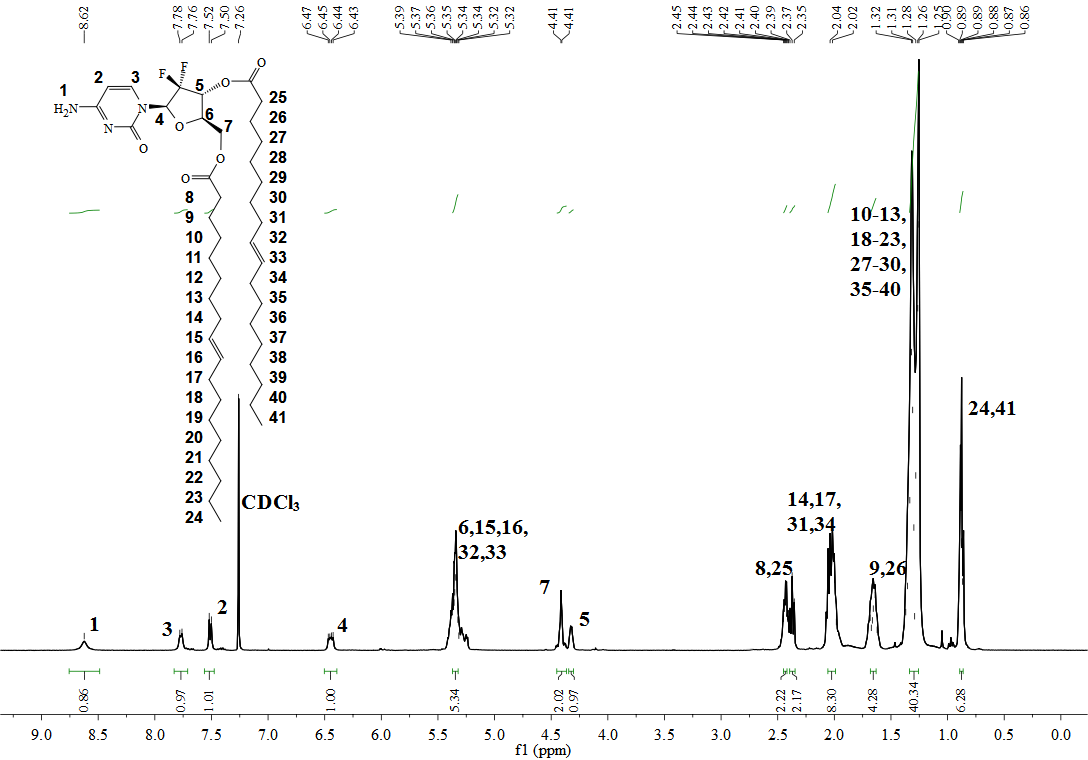


**Figure S1.** ^1^H NMR spectrum of gemcitabine in DMSO-*d*_6_ and DOG in CDCl_3_.


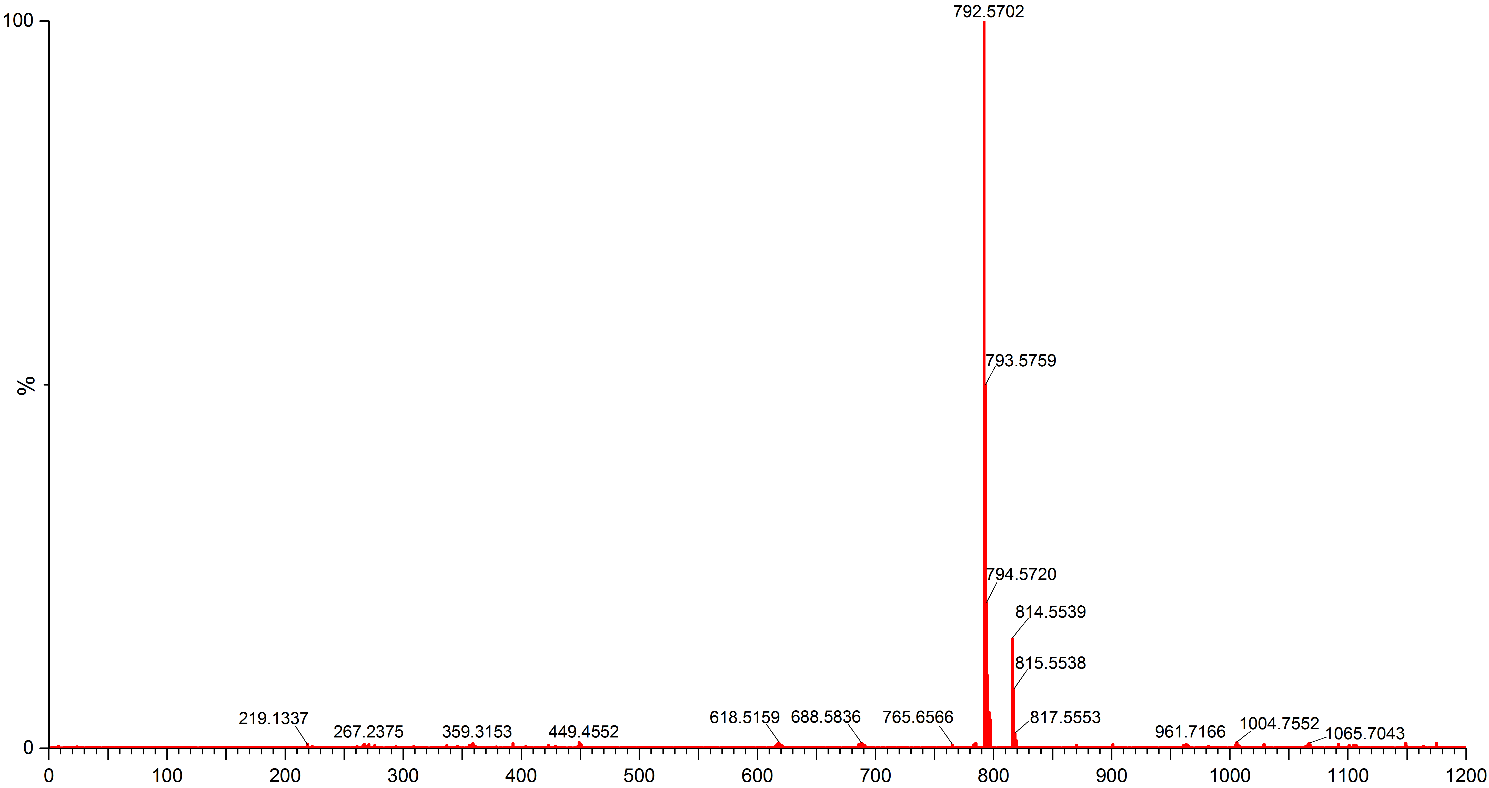


Figure S2. TOF-Q MS of DOG.


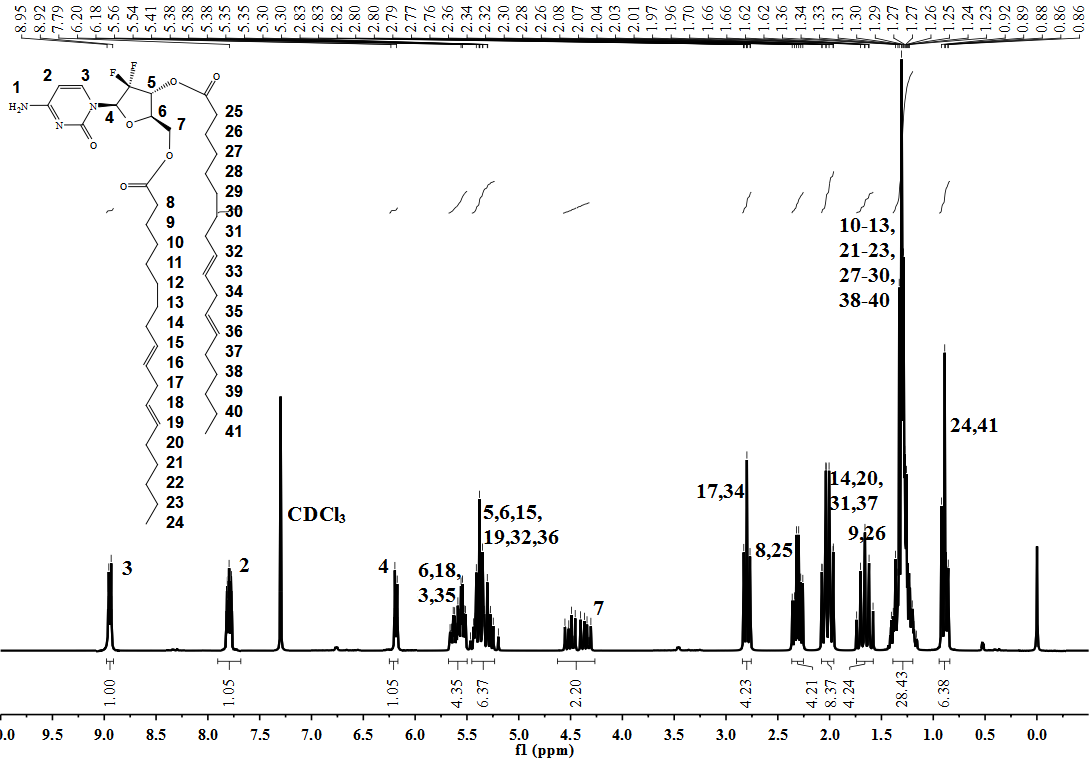


Figure S3. ^1^H NMR spectrum of DLG in CDCl_3_.


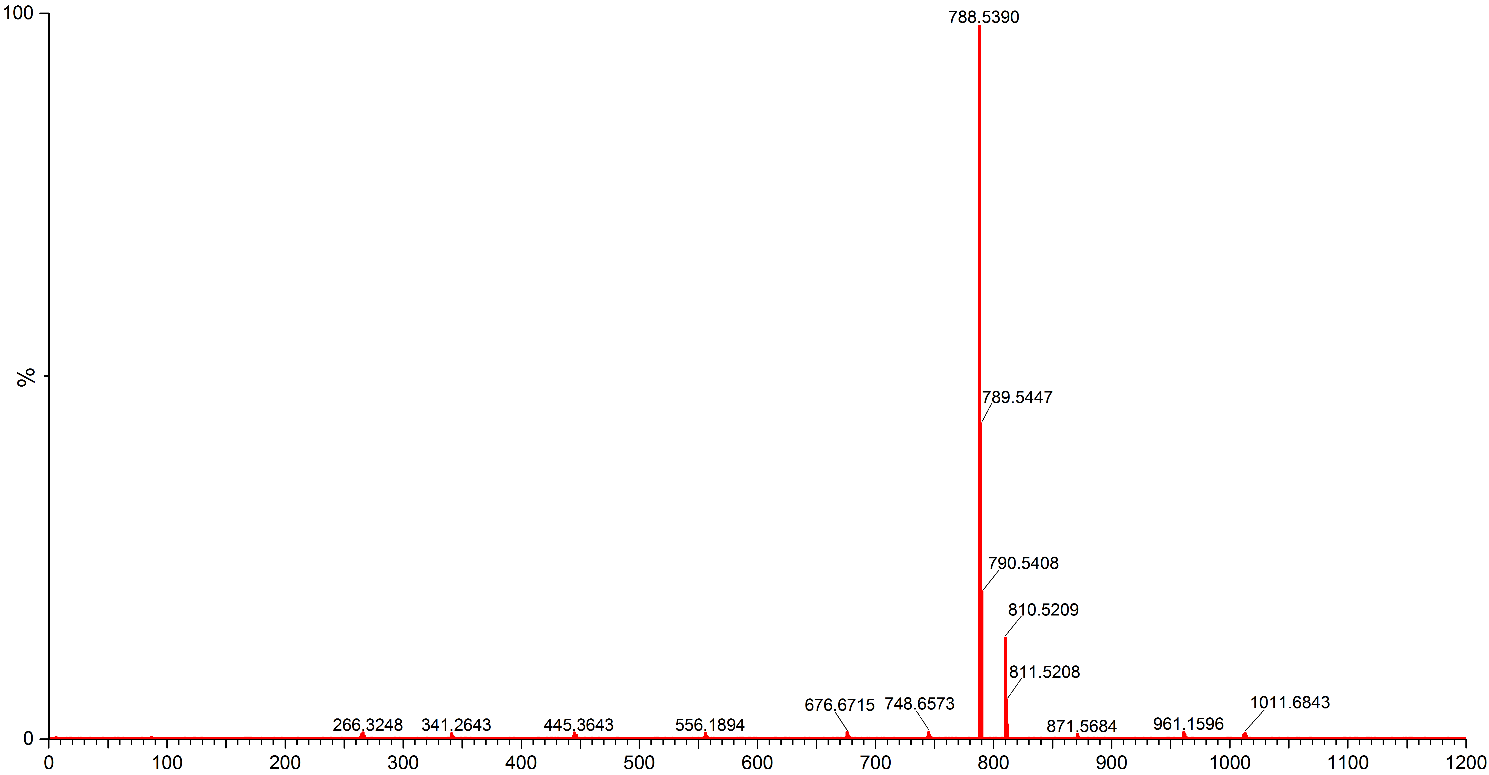


Figure S4. TOF-Q MS of DLG.


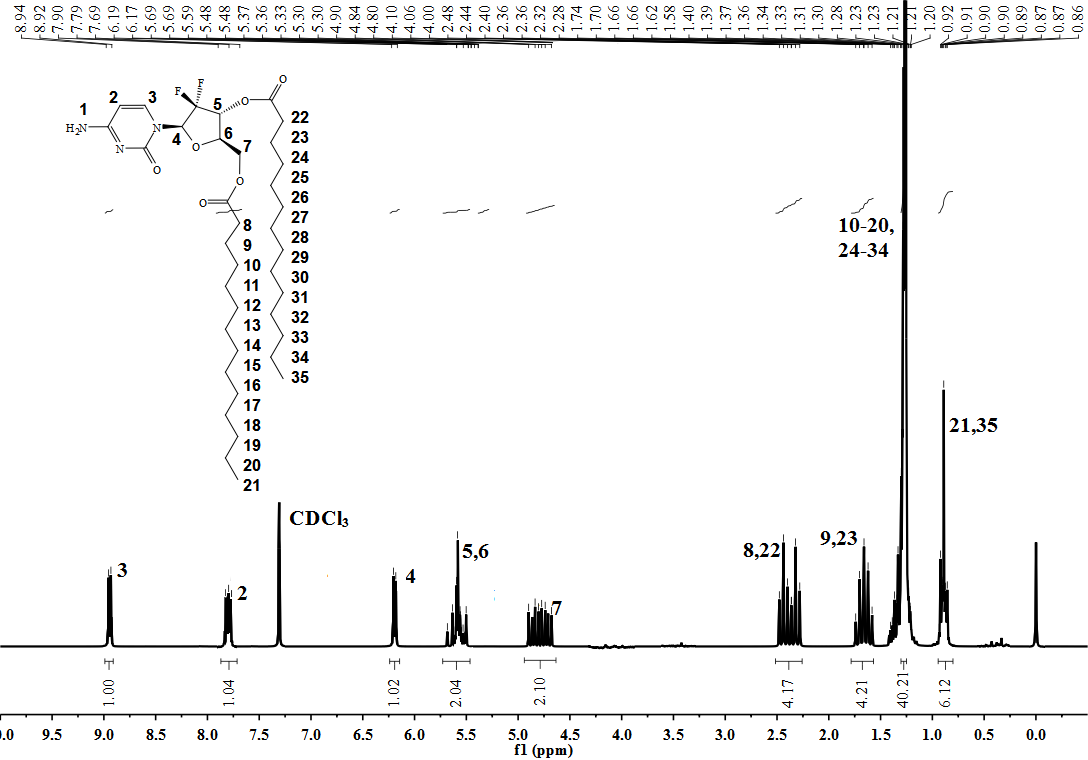


Figure S5. ^1^H NMR spectrum of DMG in CDCl_3_.


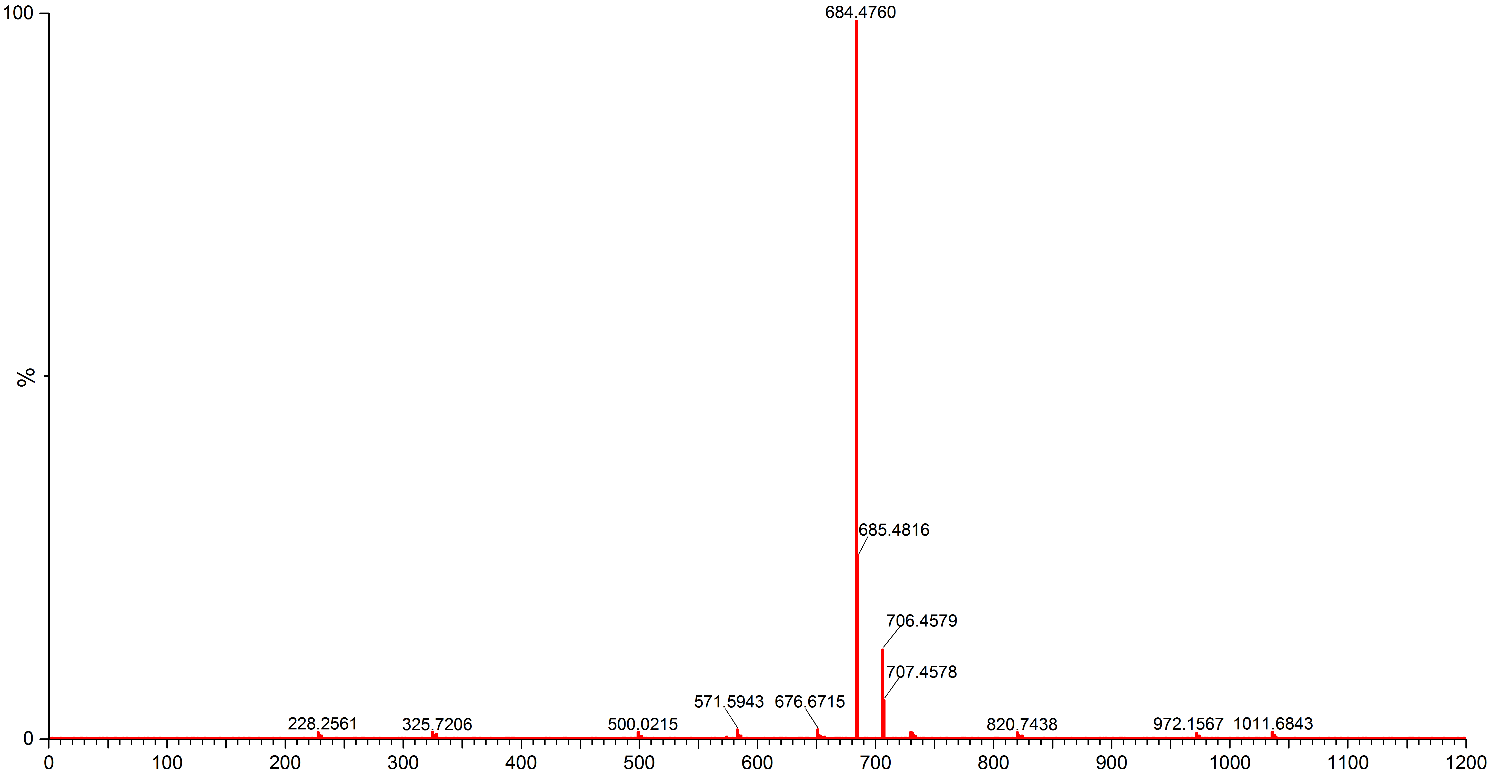


Figure S6. TOF-Q MS of DMG.


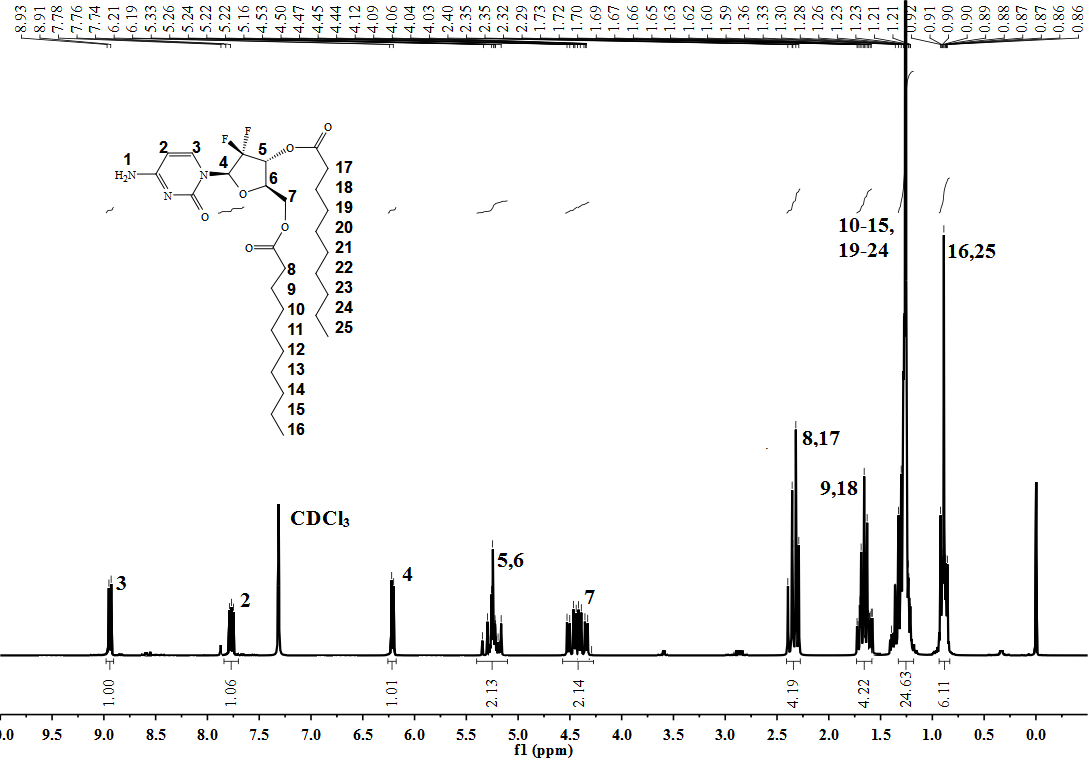


Figure S7. ^1^H NMR spectrum of DDG in CDCl_3_.


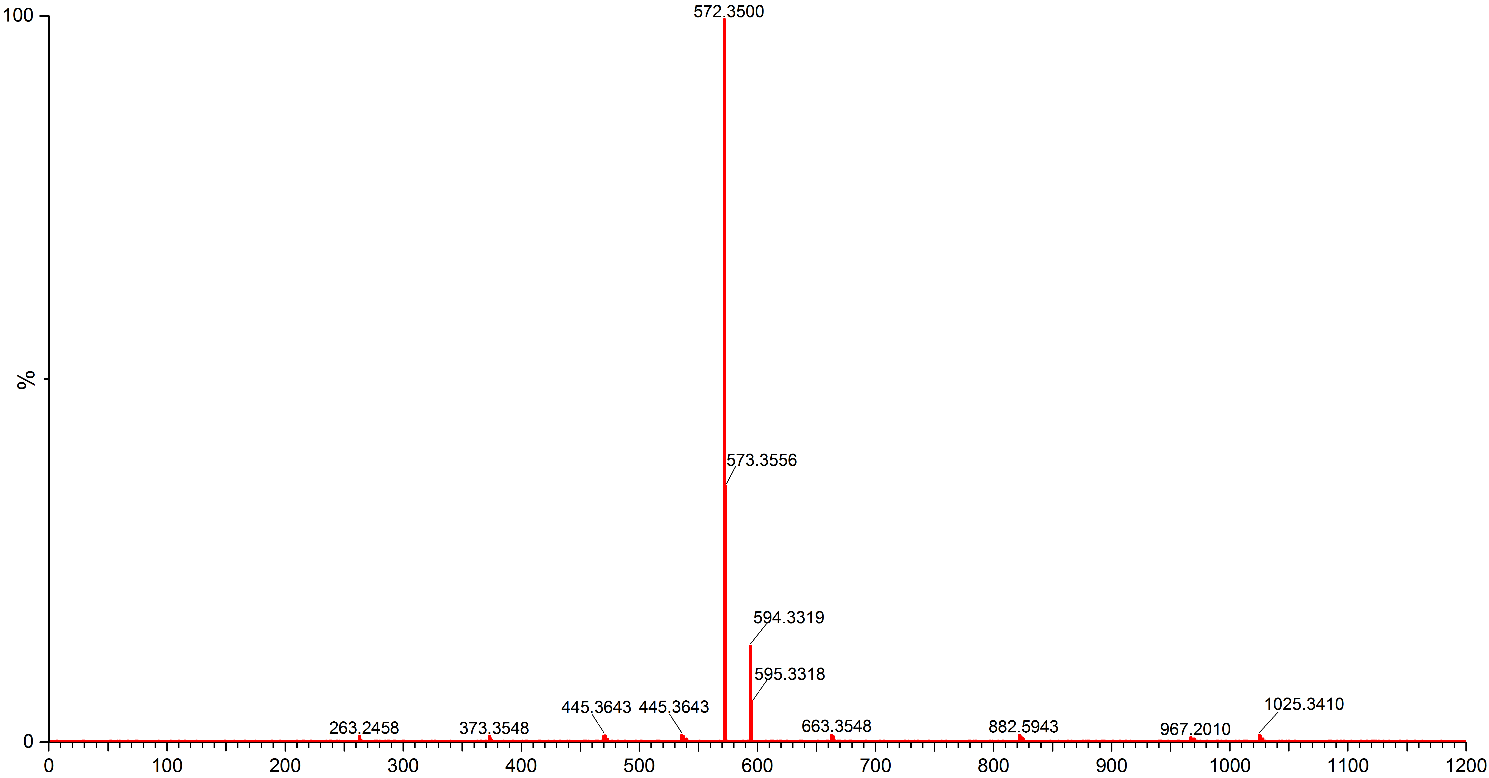


Figure S8. TOF-Q MS of DDG.


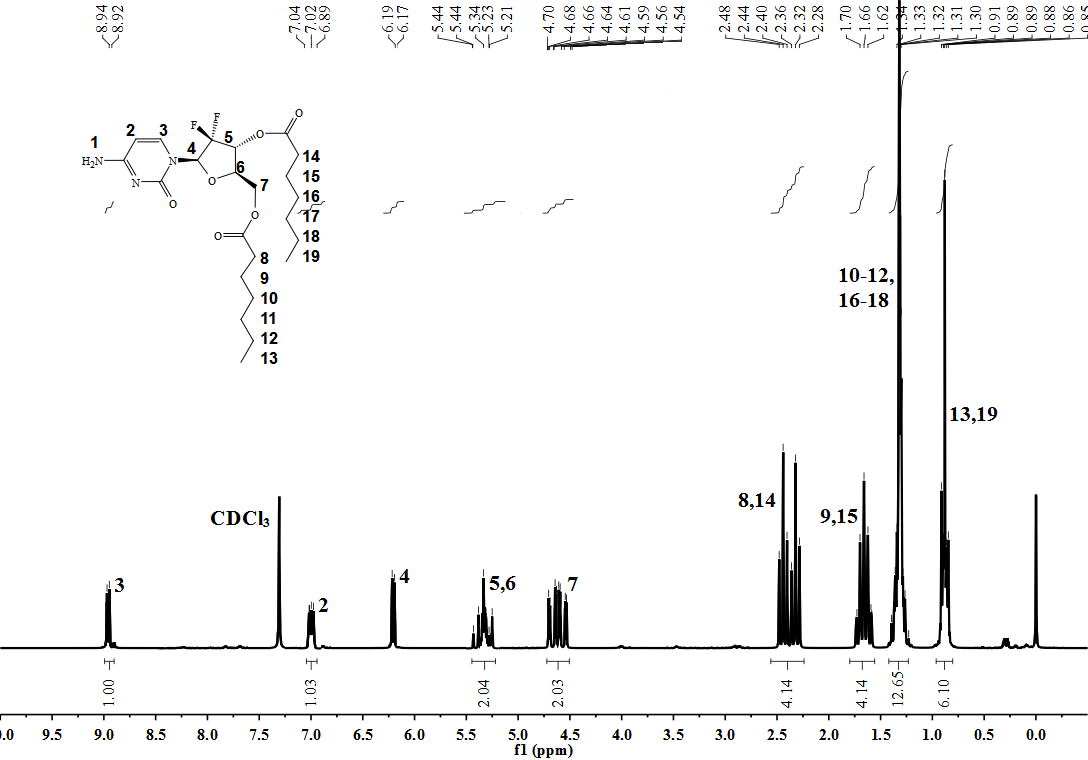


Figure S9. ^1^H NMR spectrum of DHG in CDCl_3_.


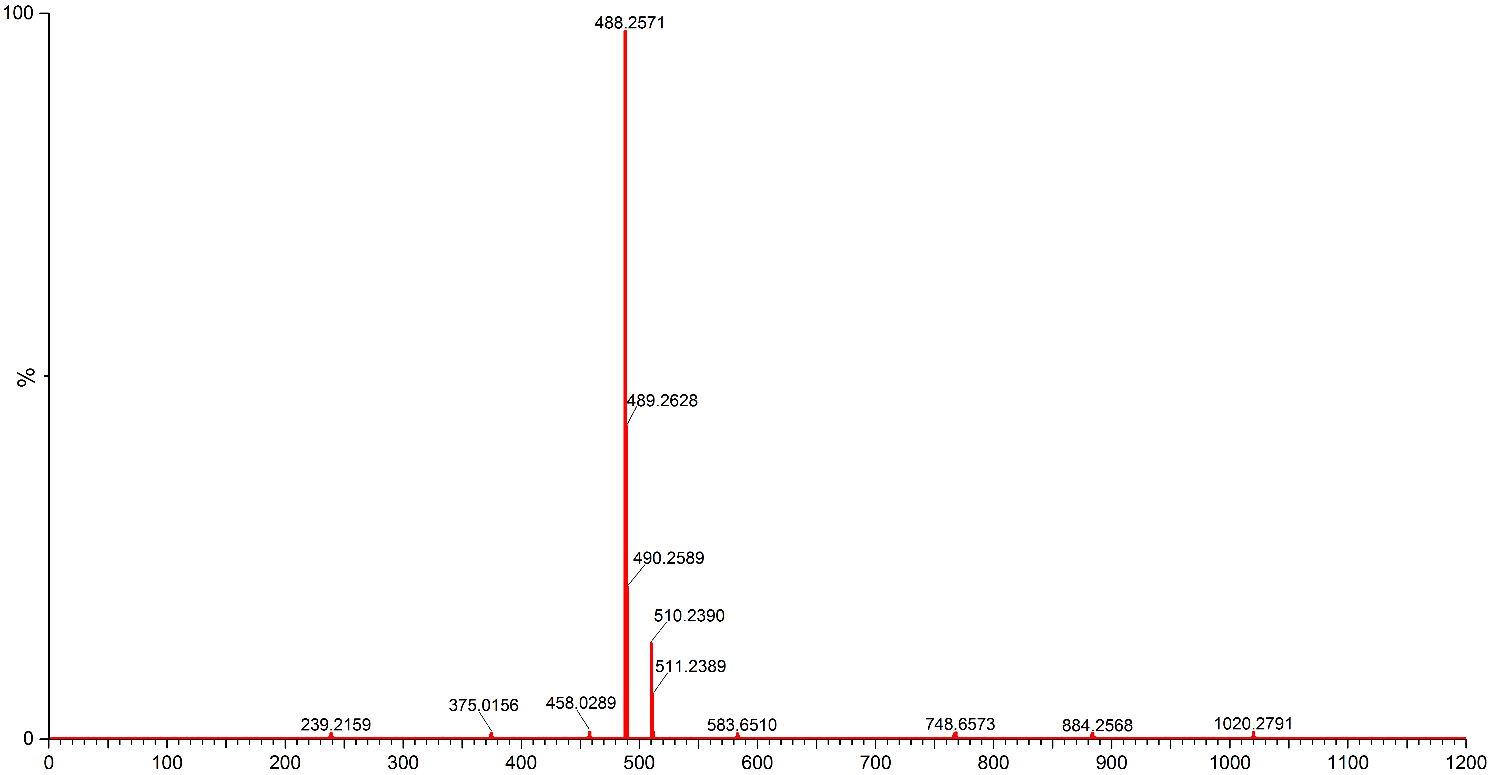


Figure S10. TOF-Q MS of DHG.


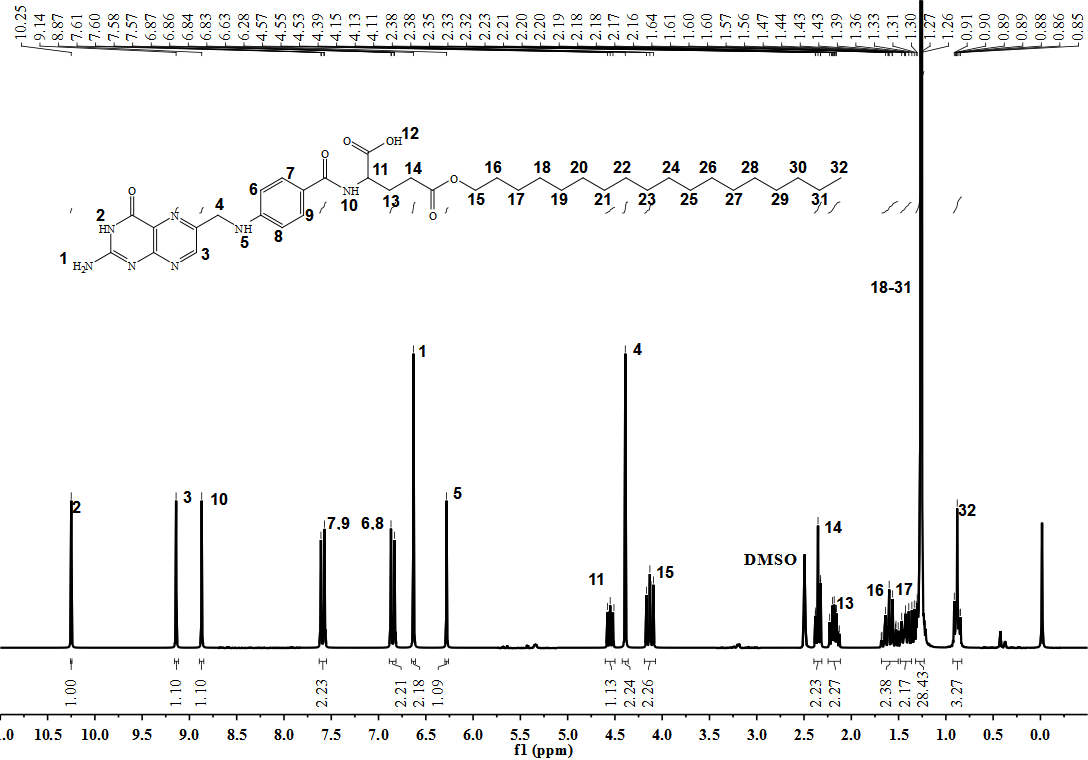


**Figure S11.** ^1^H NMR spectrum of MOFA in DMSO-*d*_6_.


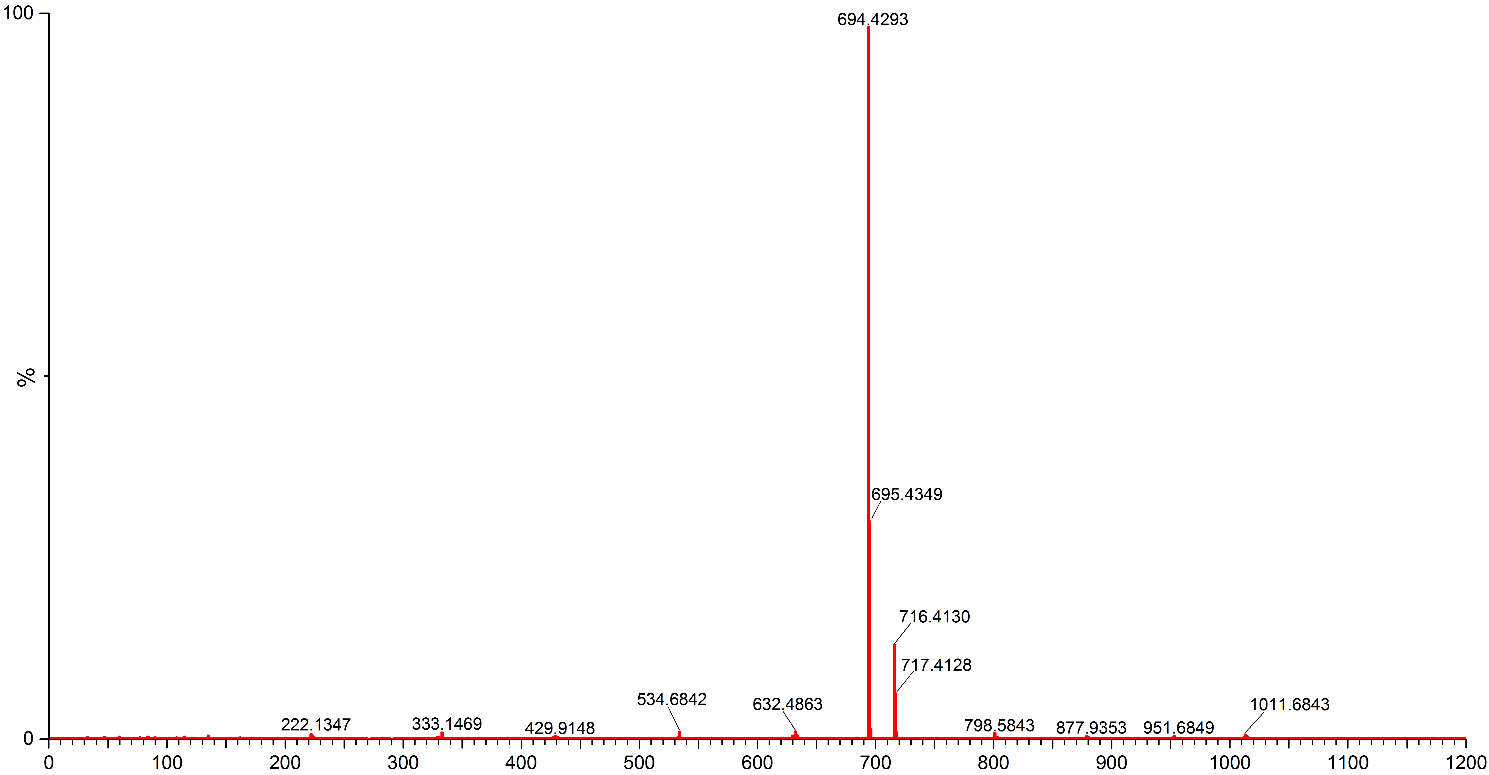


Figure S12. TOF-Q MS of MOFA.


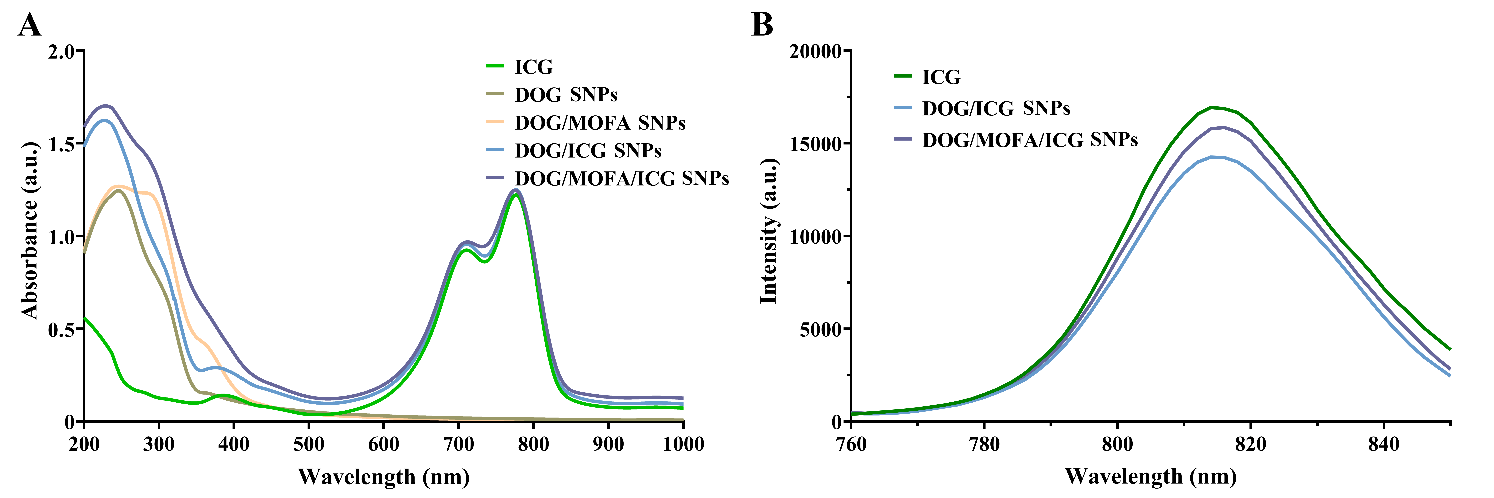


Figure S13. (A) UV-vis spectroscopy of free ICG and different nanoparticles. (**B**) Fluorescence spectra (λ_ex_= 745 nm) of free ICG, DOG/ICG and DOG/MOFA/ICG nanoparticles.


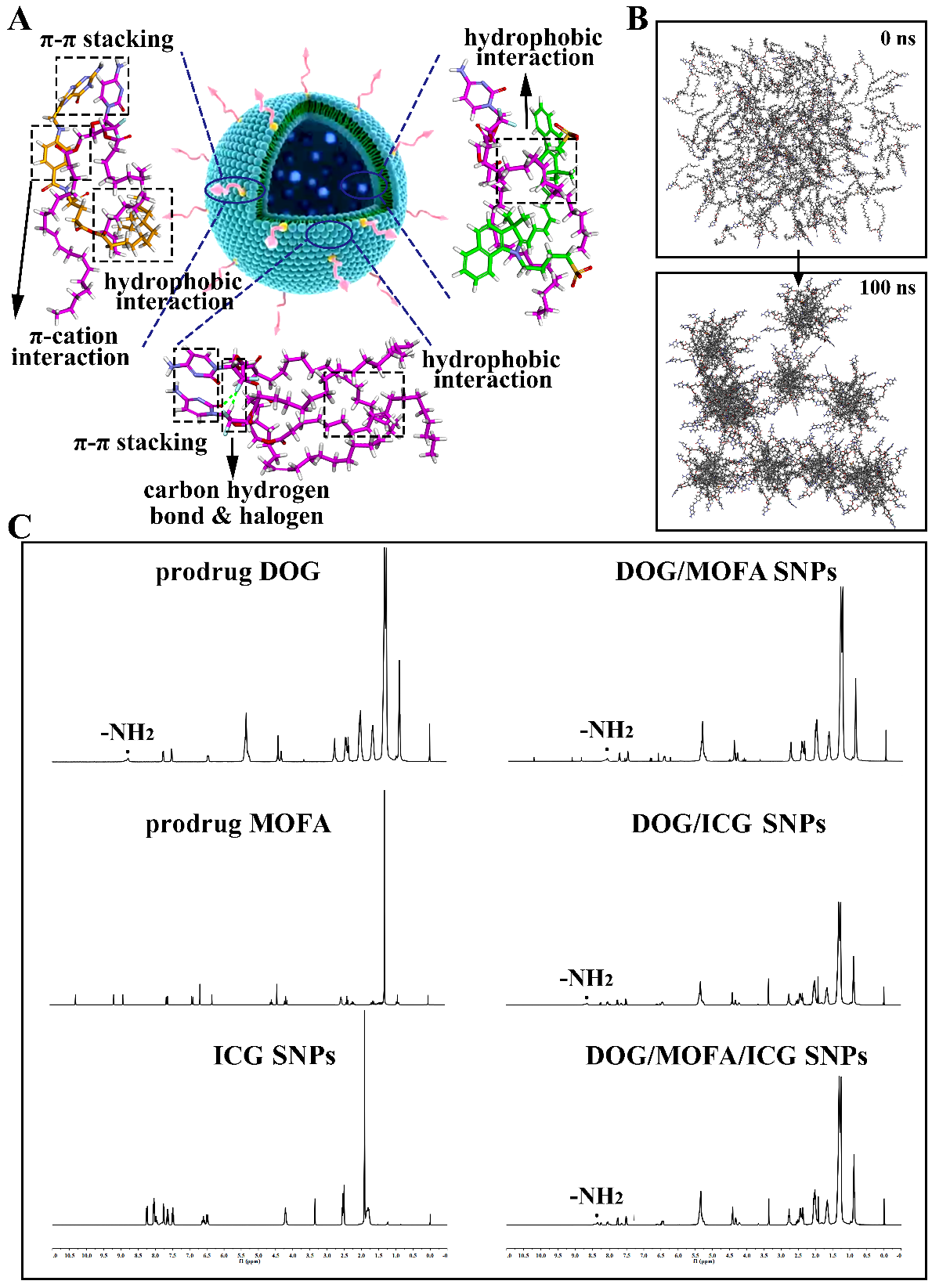


**Figure S14**. (**A**) Molecular docking result of DOG/MOFA/ICG. (**B**) Molecular structure changes of DOG, MOFA and ICG before and after simulation for 100 ns. (**C**) ^1^H NMR spectra of different prodrugs and nanoparticles.


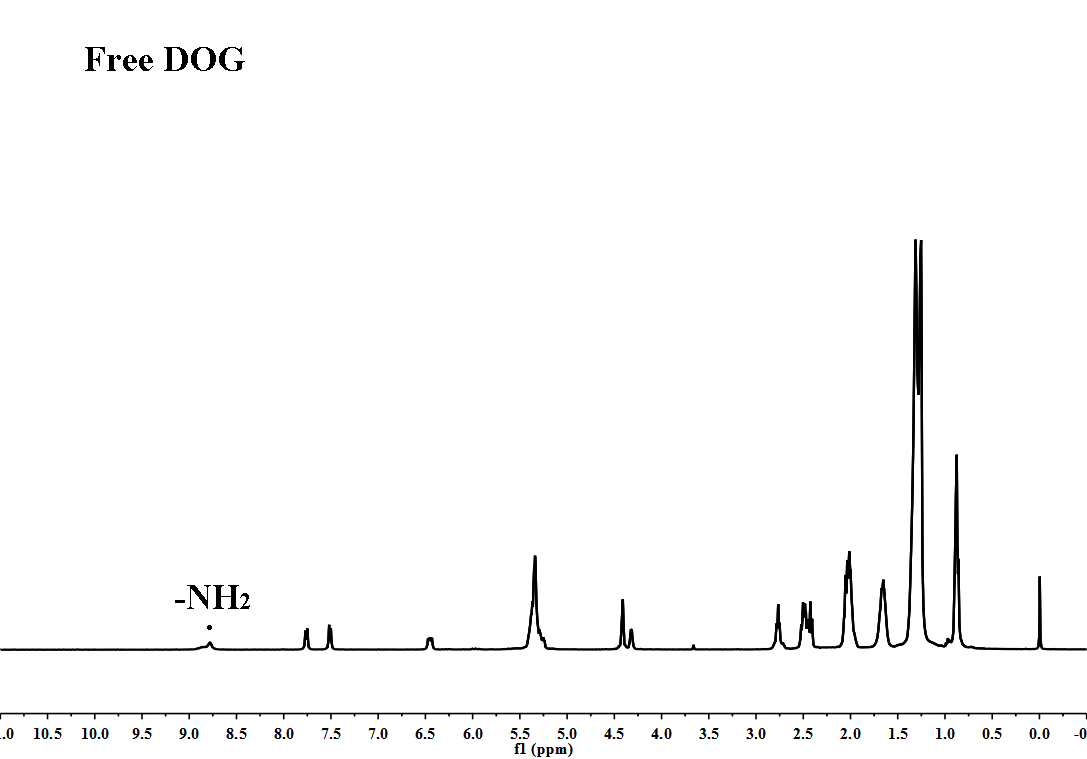


Figure S15. ^1^H NMR spectrum of free DOG in DMSO-*d*_6_.


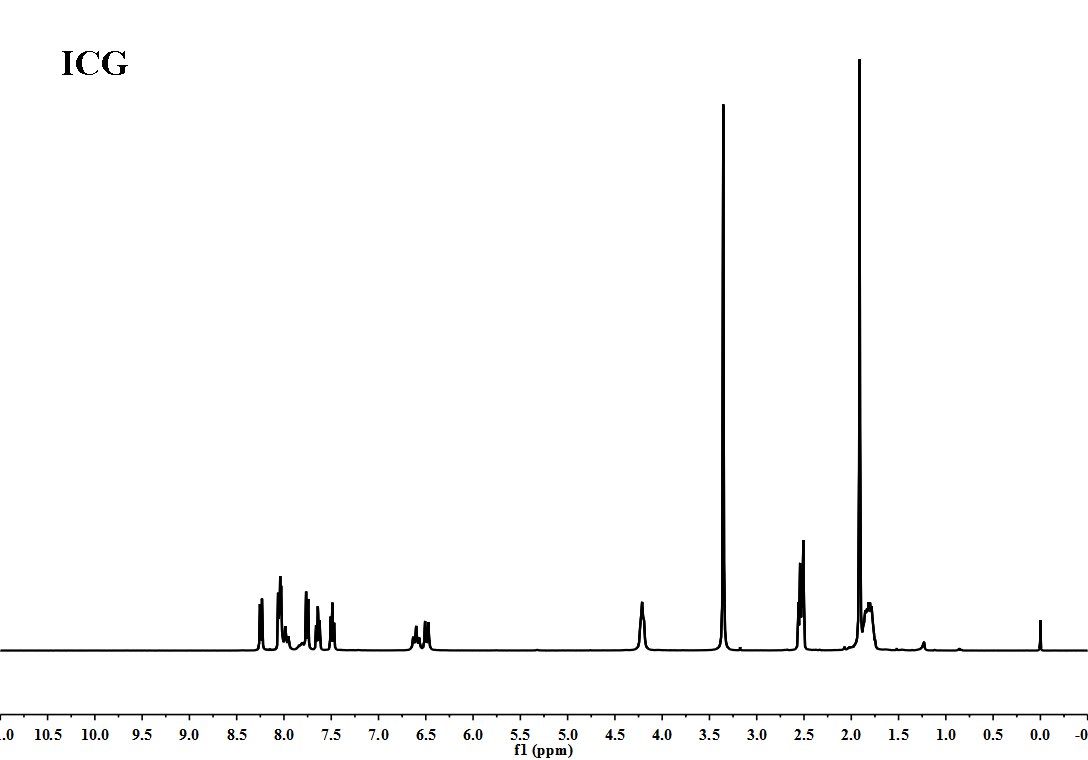


Figure S16. ^1^H NMR spectrum of ICG in DMSO-*d*_6_.


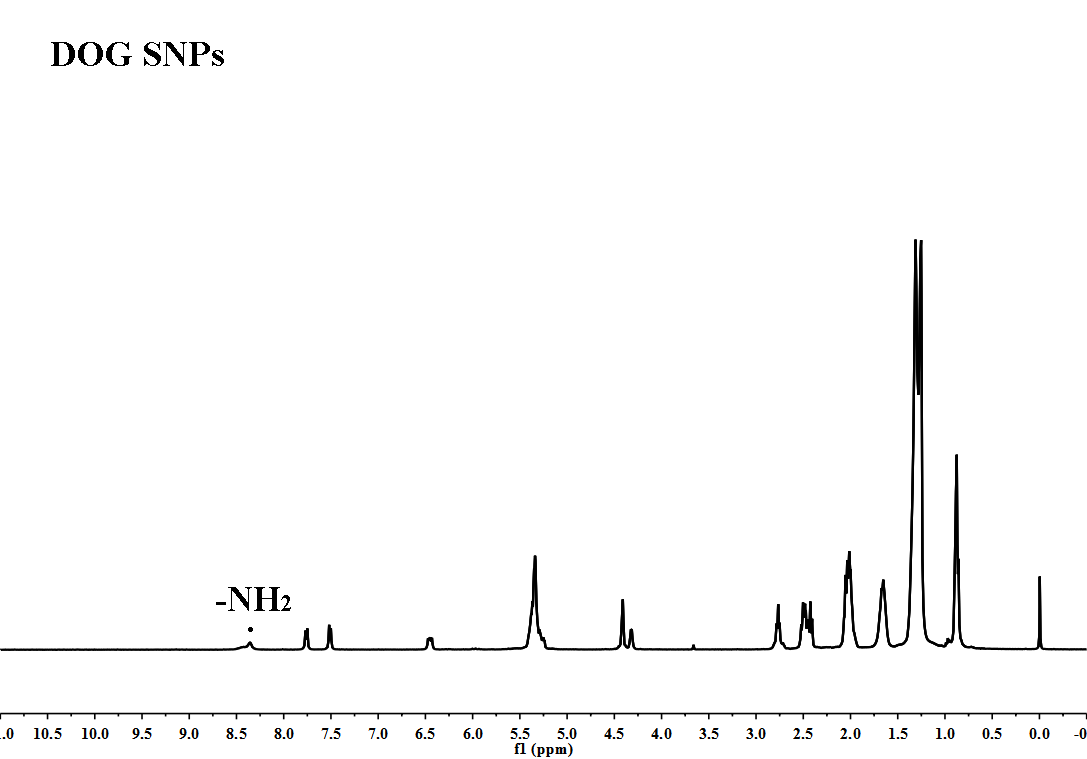


Figure S17. ^1^H NMR spectrum of DOG nanoparticles in DMSO-*d*_6_.


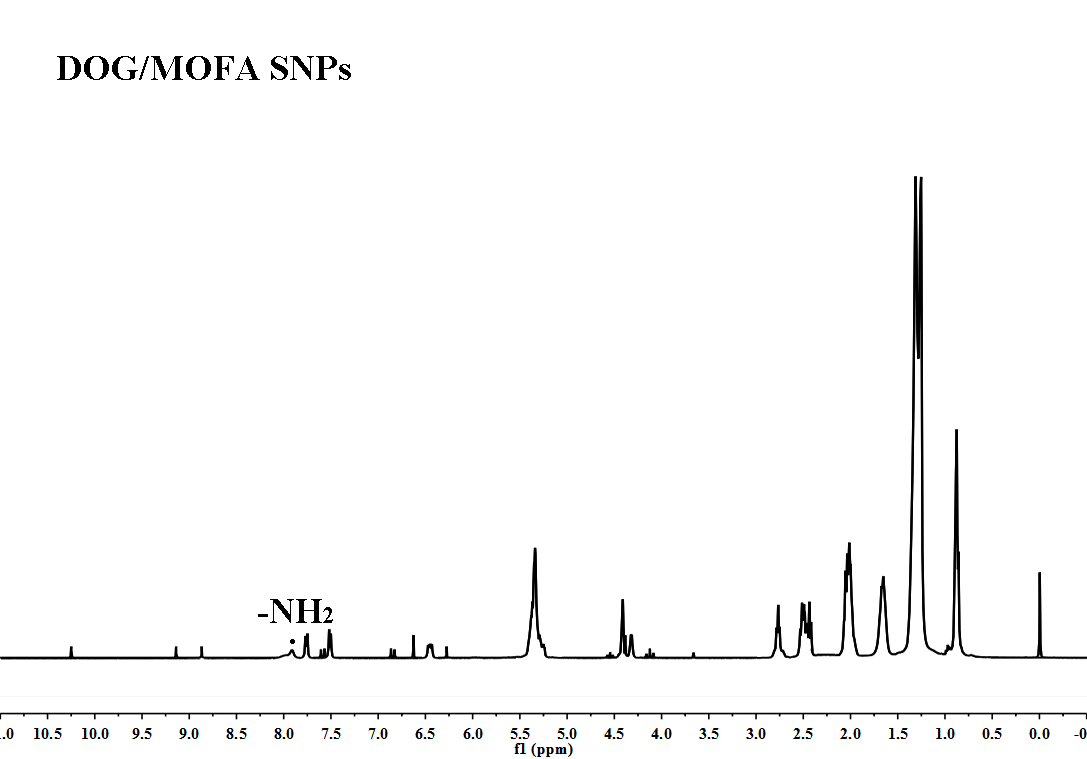


Figure S18. ^1^H NMR spectrum of DOG/MOFA nanoparticles in DMSO-*d*_6_.


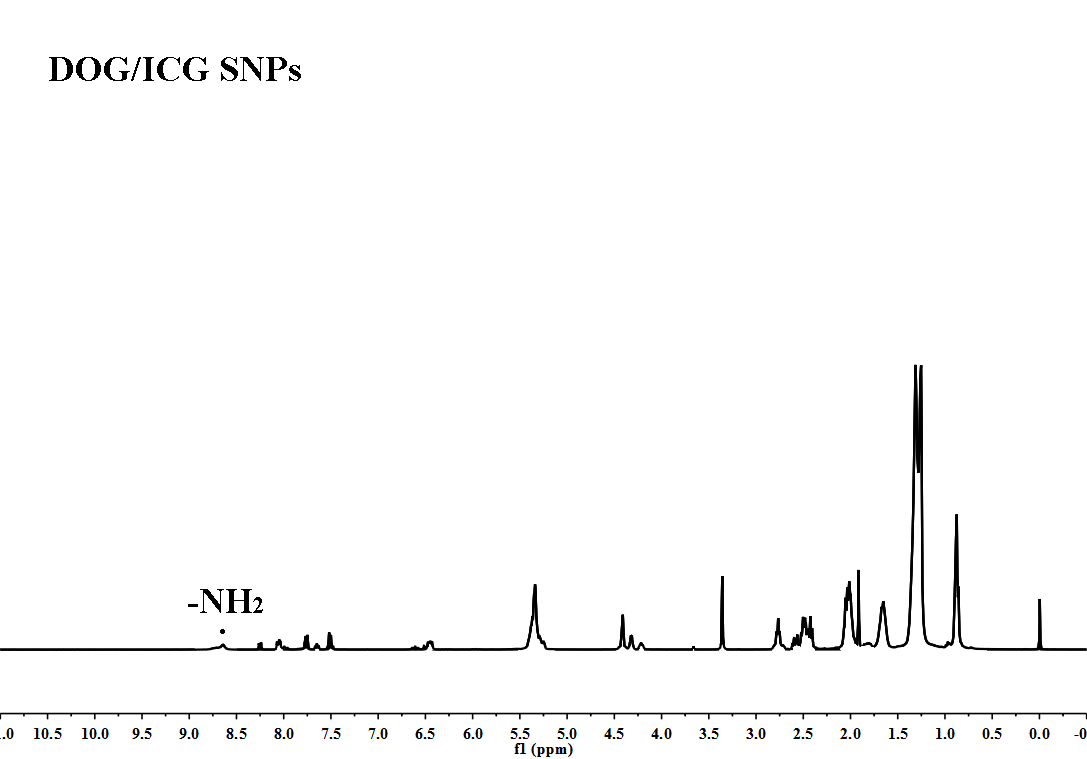


Figure S19. ^1^H NMR spectrum of DOG/ICG nanoparticles in DMSO-*d*_6_.


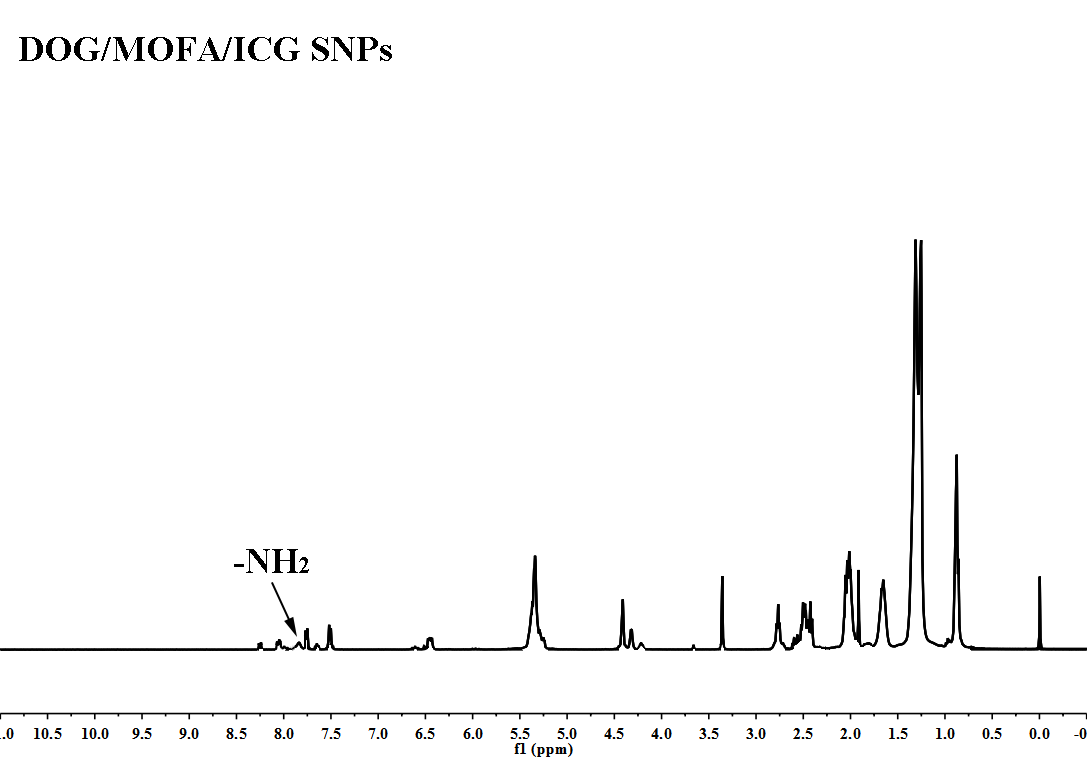


Figure S20. ^1^H NMR spectrum of DOG/MOFA/ICG nanoparticles in DMSO-*d*_6_.


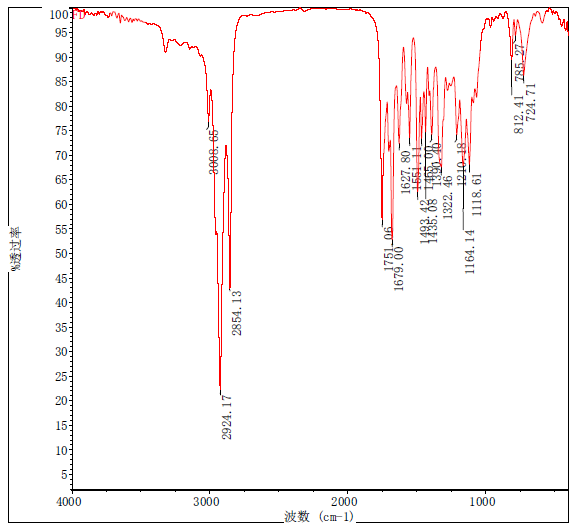


Figure S21. FT-IR spectrum of prodrug DOG.


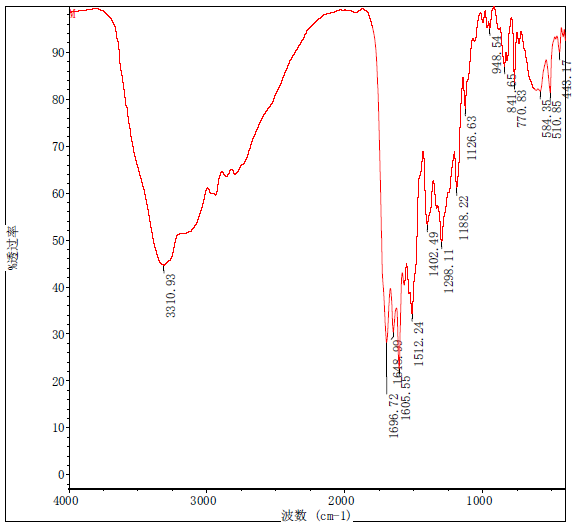


Figure S22. FT-IR spectrum of MOFA.


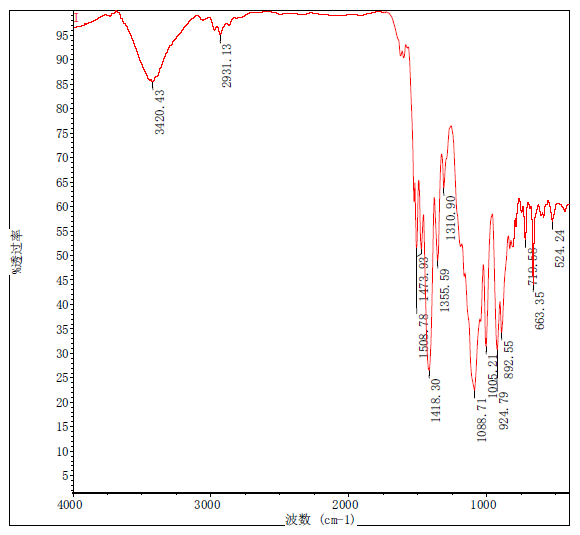


Figure S23. FT-IR spectrum of ICG.


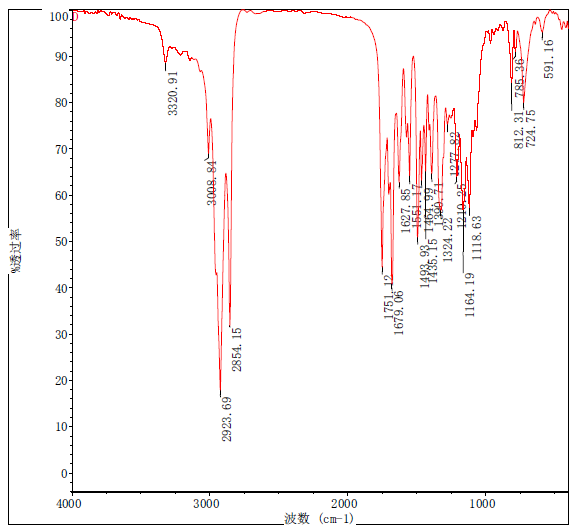


Figure S24. FT-IR spectrum of DOG nanoparticles.


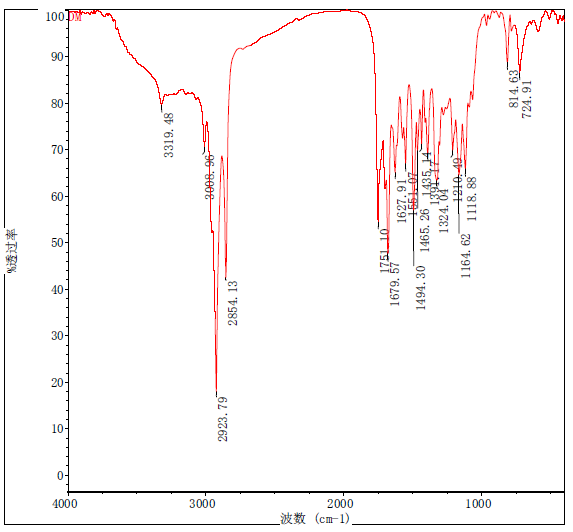


Figure S25. FT-IR spectrum of DOG/MOFA nanoparticles.


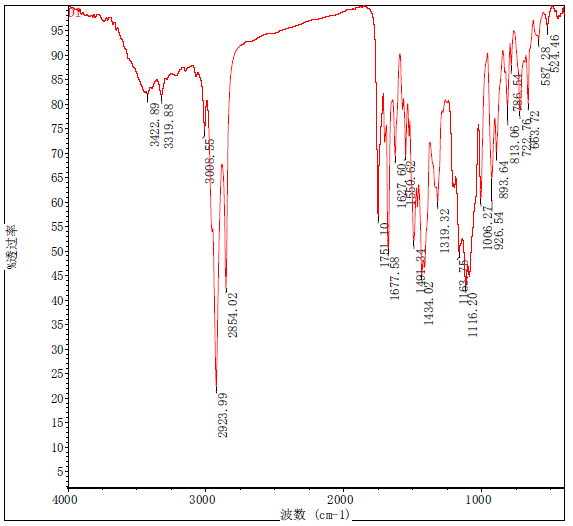


Figure S26. FT-IR spectrum of DOG/ICG nanoparticles.


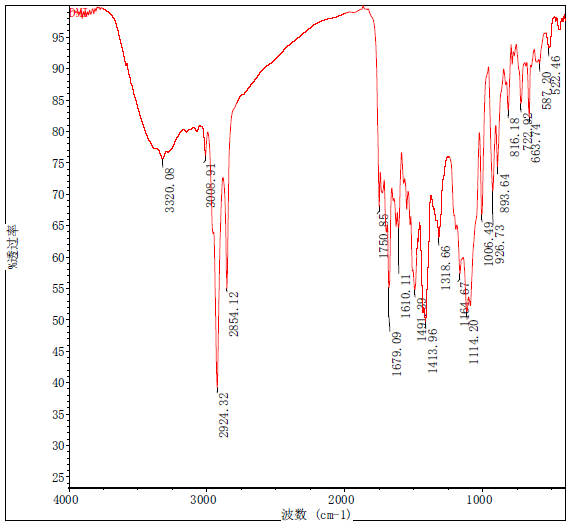


Figure S27. FT-IR spectrum of DOG/MOFA/ICG nanoparticles.


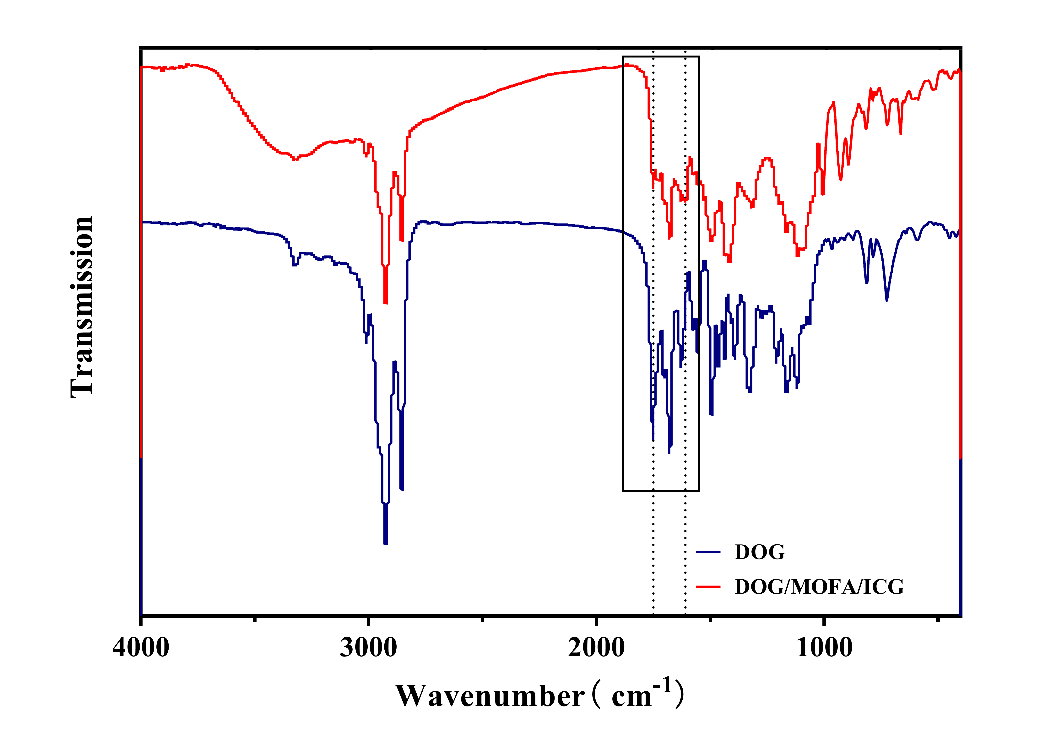


Figure S28. The overlap of FT-IR spectra of DOG and DOG/MOFA/ICG nanoparticles.


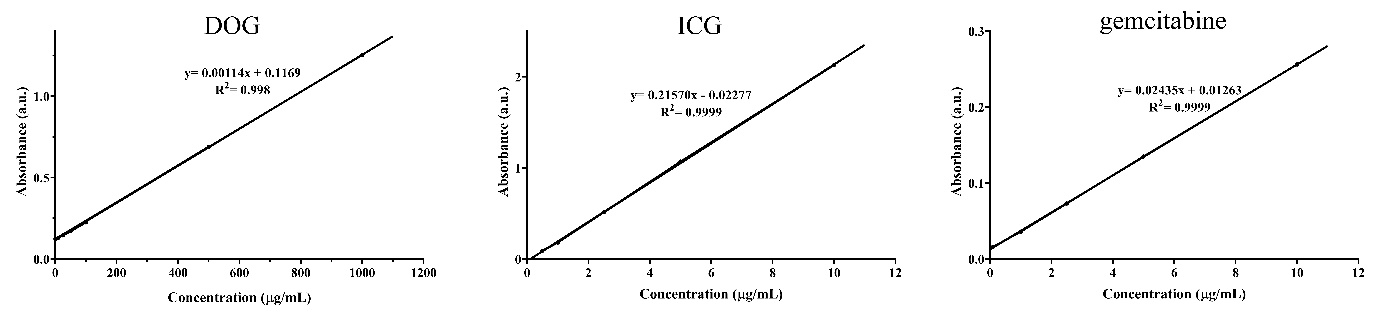


**Figure S29**. Standard curves for DOG, ICG and gemcitabine.


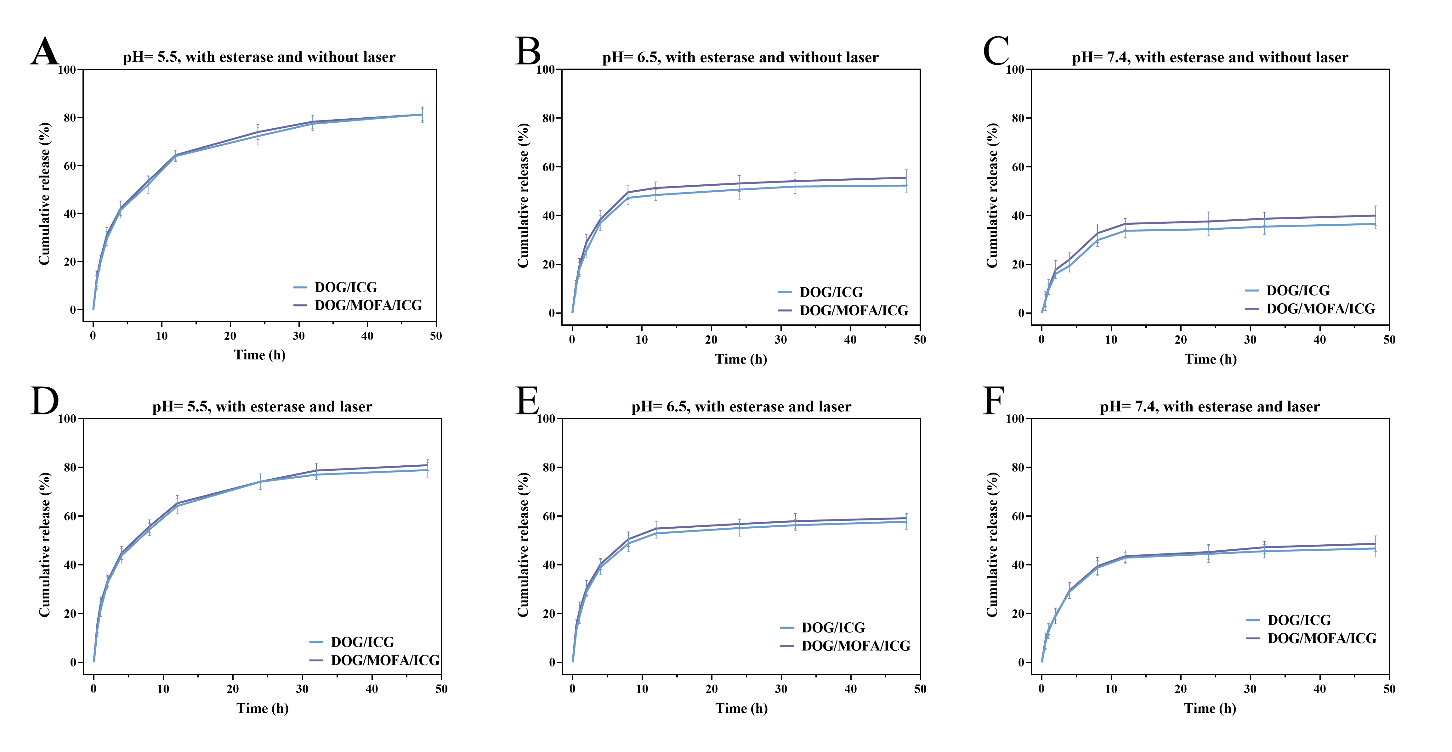


**Figure S30.** The ICG release *in vitro* of different nanoparticles without laser irradiation (**A-C**) or with laser irradiation (**D-F**) under different pH conditions with esterase (30 U/mL). Data are presented as the mean ± standard deviation (n= 3).


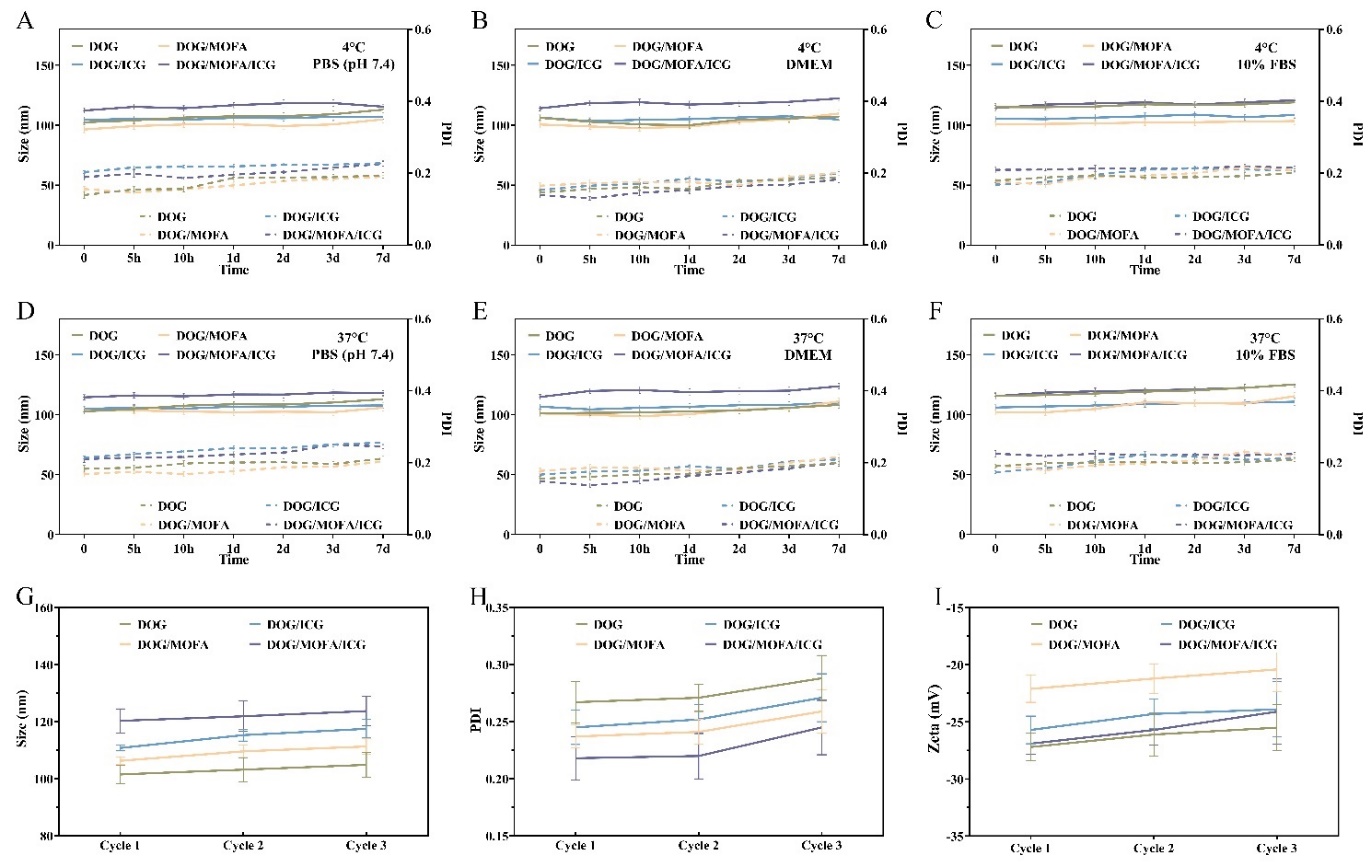


**Figure S31.** The size (solid) and PDI (dashed) of different nanoparticles in 4°C (**A–C**) and 37°C (**D–F**) within 7 days, the change of size, PDI and zeta of different nanoparticles under laser irradiation within three cycles (**G–I**). Data are presented as the mean ± standard deviation (n= 3).


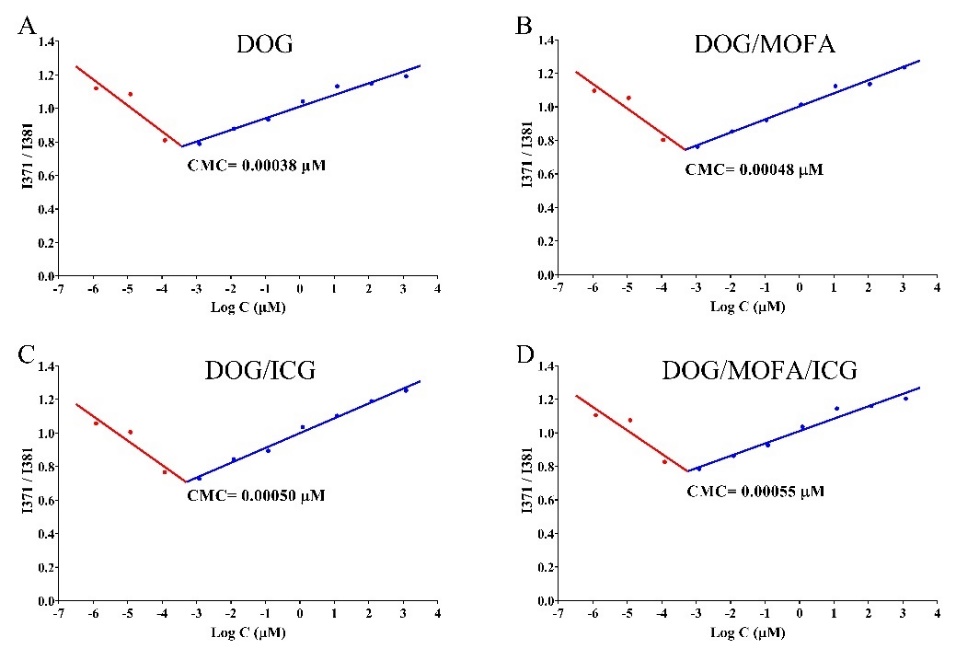


**Figure S32.** Determination of the CMC of different nanoparticles.


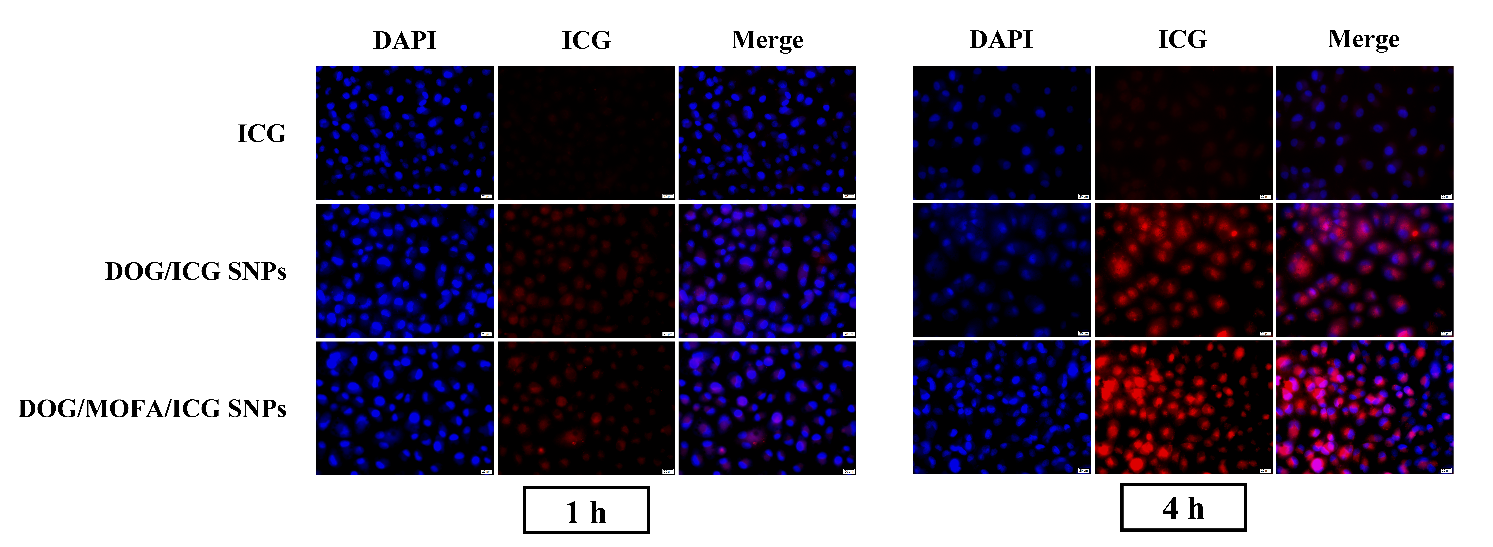


**Figure S33.** Cellular uptake images of free ICG, DOG/ICG and DOG/MOFA/ICG nanoparticles for A549 cells in 1 h and 4 h.


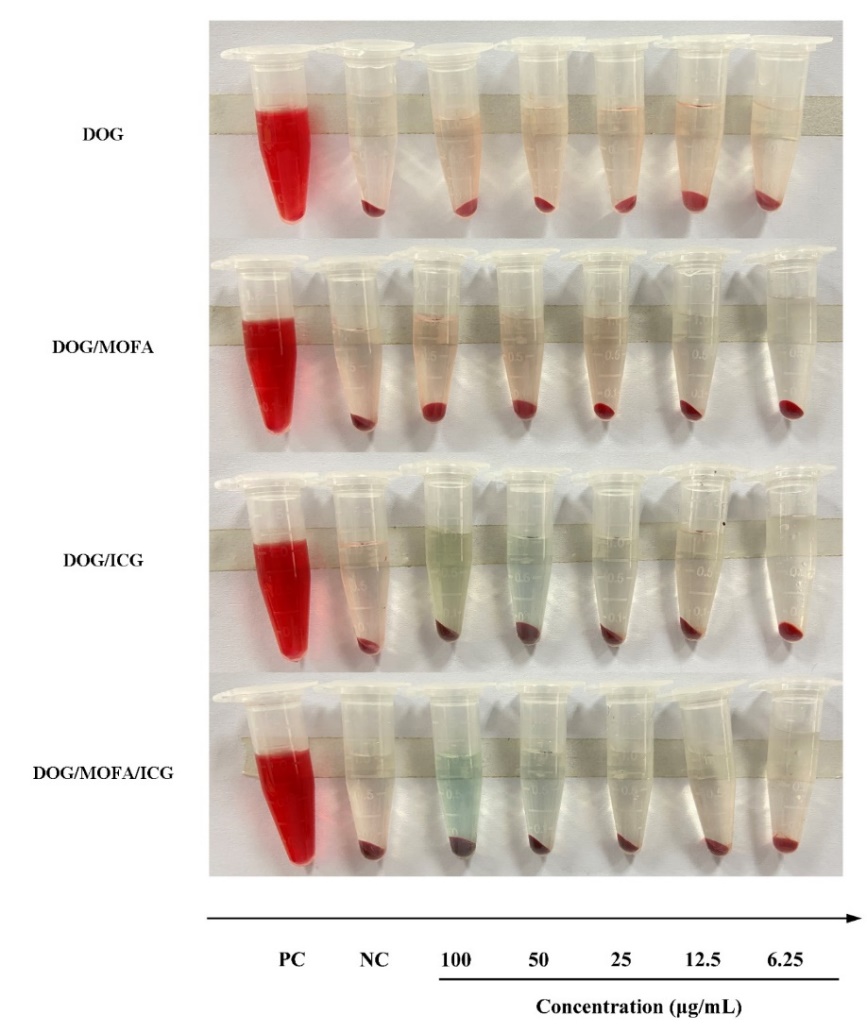


**Figure S34.** *In vitro* hemolysis percentage of different nanoparticles incubated with rabbit red blood cells at 37°C for 6 h. PBS and double distilled water incubated with RBCs were used as negative control (NC) and positive control (PC), respectively. The images on shows red blood cells treated with different nanoparticles at various concentrations at 37°C for 6 h.


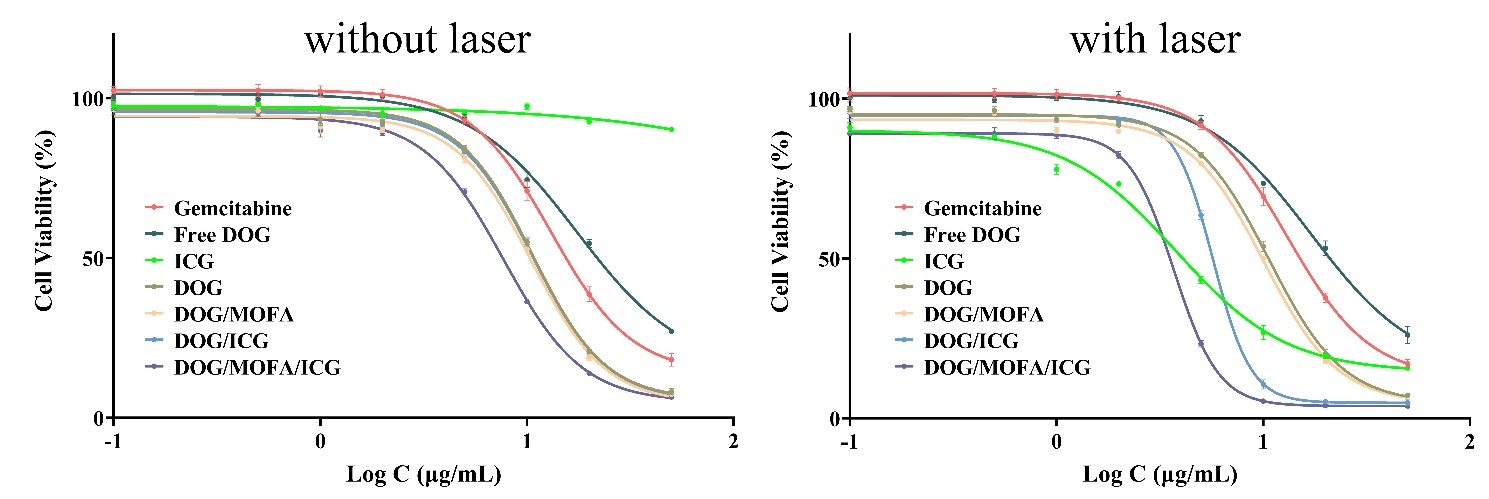


**Figure S35.** The dose-response curves of drugs and different nanoparticles against A549 cells.
